# A multi-actor stakeholder engagement progression method for co-creating and sustaining space–IoT innovation ecosystems in Africa: Lessons from the KijaniSpace project

**DOI:** 10.12688/openreseurope.23950.1

**Published:** 2026-06-04

**Authors:** Tuula-Maija Löytty

**Affiliations:** 1 Smart & Lean Hub Oy, Lahti, 15320, Finland

**Keywords:** stakeholder engagement, adaptive governance, participatory innovation, co-creation, Agricultural Innovation Systems, living labs, digital agriculture, climate-smart agriculture, innovation ecosystems, sustainability, multi-actor approach, Space–IoT integration

## Abstract

**Background:**

Stakeholder engagement is increasingly recognised as a critical component of innovation governance, sustainability and exploitation within digital agriculture and climate-smart innovation ecosystems. However, many research and innovation projects still approach stakeholder interaction primarily through dissemination-oriented activities rather than as progressively deepening participation processes supporting co-creation, collaborative ownership and long-term ecosystem continuity. This challenge is particularly relevant in AU–EU innovation ecosystems where digital agriculture solutions must operate across diverse institutional, socio-economic and environmental contexts.

**Method:**

This article proposes and operationalises a Multi-Actor Stakeholder Engagement Progression Model developed within the KijaniSpace Horizon Europe project focused on climate-smart agriculture and aquaculture in the Lake Victoria Basin. The framework conceptualises stakeholder engagement as an adaptive and iterative progression process structured around four interconnected engagement stages: Inform, Consult, Involve and Collaborate. The article combines insights from participatory innovation, Agricultural Innovation Systems, living labs and adaptive governance literature with empirical operationalisation examples from stakeholder engagement activities, workshops, communication channels and ecosystem-building processes implemented within KijaniSpace.

**Results:**

The operationalisation demonstrated that stakeholder engagement functioned not only as a dissemination mechanism, but also as a learning, co-creation and adaptive governance mechanism. Broad communication activities supported awareness and ecosystem visibility, while consultation activities generated contextual understanding and identification of operational needs and barriers. Co-creation activities strengthened participatory innovation and locally contextualised solution development, whereas collaboration-oriented activities supported ecosystem formation, trust-building, institutional dialogue and sustainability-oriented interaction. The findings further showed that stakeholder progression was dynamic rather than linear, with stakeholders moving between different participation levels according to contextual conditions, institutional roles and innovation readiness.

**Conclusions:**

The study suggests that progressively deepening stakeholder engagement can strengthen collaborative ownership, adaptive governance, sustainability and exploitation readiness within complex Space–IoT innovation ecosystems. The proposed framework provides a transferable methodological approach for connecting communication, participation, co-creation and long-term ecosystem sustainability in climate-smart agriculture and digital innovation contexts. More broadly, the article argues that stakeholder engagement should be understood not merely as dissemination support, but as a central governance mechanism through which innovation ecosystems are co-created, sustained and embedded into long-term institutional and societal structures.

## 1. Introduction

Climate-smart agriculture and digital innovation ecosystems increasingly depend on collaboration between researchers, technology developers, public authorities, SMEs, innovation intermediaries and end-users. In such environments, stakeholder engagement influences not only communication and awareness, but also innovation uptake, contextual adaptation, collaborative ownership and long-term sustainability ( Reed, 2008; World Bank, 2012). This is particularly important in innovation ecosystems that combine emerging digital technologies, environmental monitoring and local development objectives across diverse institutional and socio-economic contexts.

At the same time, many research and innovation projects still approach stakeholder engagement primarily through dissemination-oriented activities such as websites, communication campaigns, workshops and public events. Although these activities can increase visibility and awareness, they do not necessarily create long-term ownership, institutional embedding or collaborative ecosystem continuity. In many cases, stakeholder interaction remains fragmented, event-based or disconnected from co-creation, governance and sustainability processes. Consequently, communication activities may generate outreach without substantially strengthening exploitation readiness, collaborative learning or long-term innovation uptake.

These challenges become especially important in AU–EU innovation ecosystems where digital innovation must operate across diverse governance conditions, varying levels of digital readiness and highly context-specific agricultural and environmental realities. In climate-smart agriculture and aquaculture systems, the uptake of Earth Observation (EO), Internet of Things (IoT) and digital advisory solutions depends not only on technological functionality, but also on trust-building, local adaptation, institutional coordination and participatory interaction ( FAO & ITU, 2022; African Union, 2024). Previous research has similarly highlighted that innovation uptake in agricultural systems depends strongly on interaction, facilitation, co-creation and collaborative learning rather than on linear technology transfer alone ( Klerkx et al., 2010; van Ewijk & Ros-Tonen, 2021).

Recent innovation governance and participatory innovation literature increasingly emphasises that stakeholder engagement should be understood as an adaptive and iterative process rather than as a one-directional communication activity ( Reed et al., 2018). Living lab approaches, Agricultural Innovation Systems (AIS), multi-actor frameworks and human-centred design methodologies all stress the importance of involving stakeholders throughout innovation processes, from problem identification and contextual learning to co-design, experimentation and implementation ( Gardezi et al., 2024; Toffolini et al., 2023). However, despite these conceptual advances, relatively few studies operationalise how stakeholder engagement can progressively deepen from awareness-building toward collaborative ownership, sustainability and ecosystem continuity within real-life digital innovation ecosystems.

This article addresses this gap by proposing and operationalising a **Multi-Actor Stakeholder Engagement Progression Model** developed within the KijaniSpace project, a Horizon Europe initiative focused on climate-smart agriculture and aquaculture in the Lake Victoria Basin. The framework conceptualises stakeholder engagement as a dynamic and adaptive progression process structured around four interconnected engagement stages: Inform, Consult, Involve and Collaborate. Rather than treating stakeholder engagement as a separate dissemination layer, the framework positions engagement as a governance and ecosystem-building mechanism connecting stakeholder interaction, contextual learning and collaborative ecosystem development.

Using empirical operationalisation examples from KijaniSpace, the article analyses how progressively deepening stakeholder engagement can support trust-building, participatory innovation, collaborative ownership and long-term sustainability within Space–IoT innovation ecosystems. The article further explores how stakeholder progression contributes to adaptive governance, exploitation readiness, transferability and ecosystem continuity in complex multi-actor environments.

The article contributes a practical methodological framework for connecting communication, participation, adaptive governance and sustainability within digital agriculture and climate-smart innovation ecosystems. More broadly, it argues that stakeholder engagement should not be understood merely as dissemination support surrounding innovation ecosystems, but as a central mechanism through which innovation ecosystems are co-created, governed and sustained over time.

## 2. Background and conceptual context

Recent research and innovation programmes increasingly recognise that stakeholder engagement is not merely a communication activity, but a central mechanism for innovation governance, co-creation and long-term sustainability. In complex innovation ecosystems, particularly those combining digital technologies, climate services and local development objectives, one-way dissemination approaches are often insufficient to generate ownership, adoption and institutional continuity. Instead, deeper and progressively evolving stakeholder participation is required throughout the innovation lifecycle ( Reed, 2008; Reed et al., 2018).

Within European research and innovation policy, the multi-actor approach has emerged as an important framework for connecting research, practice, governance and business actors into collaborative innovation processes. The European Commission describes the approach as interactive, participatory and problem-oriented, aiming to integrate scientific, practical and local knowledge from the beginning of a project until implementation and exploitation phases ( EC: Multi-actor approach). Similarly, the EU CAP Network emphasises that innovation activities should originate from real end-user needs and be developed jointly with relevant stakeholders rather than transferred through linear dissemination models ( EU CAP Network, 2017). This perspective is especially relevant in climate-smart agriculture and digital transformation initiatives where technological solutions must be adapted to diverse socio-economic, institutional and environmental contexts.

The importance of participatory innovation is strongly reflected in agricultural innovation literature. Studies on multi-stakeholder platforms in Sub-Saharan Africa demonstrate that co-created approaches can improve productivity, institutional coordination, local learning and environmental practices. However, the same studies also highlight persistent challenges related to continuity, local ownership and donor dependency ( van Ewijk & Ros-Tonen, 2021). These findings suggest that stakeholder engagement should not be treated as a single activity or event, but rather as a dynamic progression process in which actors gradually deepen their participation and commitment over time.

Recent human-centred design and co-creation research within CGIAR and digital agriculture contexts further supports this view. Technological innovations often fail when designed primarily from technological or research-driven perspectives without sufficient integration of user realities, local capacities and institutional conditions. The so-called “design–reality gap” remains a common barrier in agricultural digitalisation initiatives ( Müller et al., 2024). Consequently, participatory approaches increasingly seek to move stakeholders from passive information recipients toward active problem definers, co-designers and implementation partners.

Living lab literature provides an operational perspective on how such participation can be organised in practice. Living labs are generally understood as real-life experimentation and co-creation environments where users, researchers, businesses and public actors collaboratively develop and test innovations in authentic contexts. In agriculture, living lab approaches have been operationalised through interviews, needs assessments, co-design workshops, pilot demonstrations, field experimentation and iterative feedback mechanisms ( Gardezi et al., 2024; Toffolini et al., 2023). Importantly, these approaches acknowledge farmers’ experiential and contextual knowledge as a legitimate and necessary component of innovation development.

Agricultural Innovation Systems (AIS) literature adds a broader governance perspective to stakeholder engagement. According to the World Bank, innovation emerges not only from research investments or technological advancements, but also from interaction, coordination, institutional support and collective learning among multiple actors ( World Bank, 2012). Innovation networks therefore require facilitation mechanisms, adaptive governance structures and intermediary functions capable of connecting different actor groups and translating knowledge across institutional boundaries ( Klerkx et al., 2010). From this perspective, stakeholder engagement becomes part of innovation system governance rather than a separate dissemination function.

The maturity of stakeholder participation has been conceptualised in several participation frameworks. One of the most widely used is the IAP2 Spectrum of Public Participation, which describes participation as progressing from informing and consulting toward involving, collaborating and empowering stakeholders ( IAP2 Federation, n.d.). Although originally developed for public participation contexts, the framework provides a useful conceptual basis for understanding how engagement depth can evolve in innovation ecosystems. Reed and colleagues further argue that successful participation depends on contextual adaptation, trust-building, legitimacy of knowledge, institutional conditions and the quality of interaction processes ( Reed, 2008; Reed et al., 2018). Participation is therefore not static or linear, but iterative and adaptive.

In African digital agriculture contexts, these dimensions become particularly important. FAO and ITU analyses show that digital agriculture readiness in Sub-Saharan Africa is shaped by infrastructure availability, policy frameworks, institutional capacities, digital literacy, business ecosystems and local innovation support structures ( FAO & ITU, 2022). At the same time, the African Union Digital Agriculture Strategy emphasises co-creation, local entrepreneurship and collaborative ecosystem building as prerequisites for sustainable digital transformation ( African Union, 2024). Studies on climate information services additionally demonstrate that trust, local language adaptation, social networks and participatory interaction significantly influence whether farmers actually adopt and use digital services in practice ( Appiah et al., 2025).

These insights suggest that effective stakeholder engagement in Space–IoT innovation ecosystems requires more than communication and awareness raising. It requires mechanisms that progressively deepen participation, strengthen trust, support collaborative ownership and embed innovations into local institutional and business ecosystems. Consequently, stakeholder engagement can be understood as a dynamic innovation governance mechanism that connects communication, consultation, co-creation, experimentation, exploitation and sustainability into a continuous progression process.

## 3. KijaniSpace context

KijaniSpace is a Horizon Europe innovation project that explores how space-based services and digital technologies can support climate-smart agriculture and aquaculture in the Lake Victoria Basin in East Africa ( KijaniSpace website). The project operates across Kenya, Uganda and Tanzania, addressing challenges related to food production, environmental sustainability, water management, climate variability, and access to digital services in rural and peri-urban areas.

The Lake Victoria Basin is one of Africa’s most important socio-economic regions, supporting millions of people through farming, fisheries, aquaculture and related value chains ( LVBC website). At the same time, the region faces increasing pressure from climate change, environmental degradation, declining fish stocks, fragmented advisory services, and limited access to timely information for decision-making ( FAO, 2022; IPCC, 2022). Smallholder farmers, fishers and local enterprises often lack affordable digital tools that could improve productivity, resilience and resource efficiency.

KijaniSpace addresses these challenges by combining Copernicus Earth Observation data with Internet of Things (IoT) technologies and local digital innovation ecosystems. The project develops and demonstrates practical applications that integrate satellite data, environmental monitoring, sensor technologies and digital advisory services. The overall approach seeks to improve the accessibility and usability of space-based information for local stakeholders and end-users.

A central operational concept within the project is the KijaniBox (also referred to as SpaceIoTBox), which represents a modular and adaptable integration environment for space and IoT solutions. The concept supports experimentation, local innovation and deployment of digital services tailored to regional needs. The approach enables the combination of Earth Observation data, environmental sensing, connectivity solutions and user-oriented applications in agriculture and aquaculture contexts. Demonstration activities include use cases related to crop monitoring, water and environmental observations, aquaculture support, and climate-related decision-making.

The KijaniSpace framework combines European and African partners representing research organisations, SMEs, innovation hubs, public authorities, regional organisations and technical experts. The structure supports interdisciplinary collaboration between Earth Observation specialists, IoT developers, agricultural and aquaculture stakeholders, innovation intermediaries and policy actors. The project therefore operates not only as a technology initiative, but also as a multi-actor innovation ecosystem connecting technical development, local capacity building and stakeholder engagement.

Within this broader innovation ecosystem, particular emphasis is placed on stakeholder interaction, knowledge exchange, ecosystem building, replication pathways and long-term sustainability of project results. The project applies a multi-actor approach that combines communication, participatory engagement, co-creation activities, innovation support mechanisms and partnership development across regional and international levels. These activities aim to strengthen awareness, local ownership, innovation uptake and collaboration between African and European stakeholders while supporting the long-term usability, scalability and sustainability of the developed solutions. In this context, stakeholder engagement is not treated solely as a dissemination activity, but as a governance and ecosystem-building mechanism that supports experimentation, collaboration and future exploitation opportunities.

## 4. Methodological framework: Multi-Actor Stakeholder Engagement Progression Model

The Multi-Actor Stakeholder Engagement Progression Model was developed to support the co-creation, governance, uptake and long-term sustainability of Space–IoT innovation ecosystems in climate-smart agriculture and aquaculture contexts. The framework combines principles from participatory innovation, multi-actor approaches, Agricultural Innovation Systems (AIS), living lab methodologies and adaptive innovation governance ( Klerkx et al., 2010; Reed, 2008; Reed et al., 2018). It conceptualises stakeholder engagement not as a one-way dissemination activity, but as a dynamic and iterative progression process through which stakeholders gradually deepen their participation, interaction, ownership and collaborative commitment.

The framework is based on the assumption that sustainable innovation ecosystems require more than technological development alone. Long-term uptake, scalability and institutional continuity depend on how effectively different stakeholder groups are engaged throughout the innovation process. Consequently, the model treats stakeholder engagement as a governance and ecosystem-building mechanism that connects progressively deepening forms of stakeholder interaction into a continuous progression pathway ( World Bank, 2012; EC: Multi-actor approach).

The framework organises stakeholder participation into four interconnected engagement stages:

1. Inform
2. Consult
3. Involve
4. Collaborate

The four stages represent progressively increasing levels of participation, trust, interaction intensity, resource commitment and stakeholder ownership. At the same time, the model recognises that stakeholder engagement is not strictly linear. Stakeholders may progress gradually, pause, regress, or participate simultaneously across multiple engagement levels depending on contextual conditions, institutional maturity, digital readiness, perceived relevance and ecosystem dynamics ( Reed et al., 2018).

The framework further assumes that different engagement stages generate different forms of value. Broad communication-oriented activities can efficiently increase awareness and visibility among large stakeholder groups, while deeper collaborative activities involve fewer participants but generate stronger ownership, trust, institutional embedding and sustainability potential. The model therefore balances broad outreach with progressively deeper participation mechanisms.

The methodological logic of the framework can be summarised as progressive stakeholder deepening:

**Inform → Consult → Involve → Collaborate**

The framework should not be interpreted as a linear stakeholder funnel, but as an adaptive ecosystem in which stakeholders may move iteratively across different engagement levels depending on contextual conditions, institutional roles and innovation readiness.

The framework assumes that progressively deepening engagement supports participation, co-creation, ownership and long-term sustainability.

The framework is underpinned by six interconnected methodological principles that together support adaptive stakeholder progression and innovation ecosystem development.

### 4.1 Progressive engagement depth

The first principle is progressive engagement depth. The framework assumes that stakeholder participation evolves gradually through increasing levels of interaction, trust, commitment and ownership. Initial engagement activities may focus on awareness raising and information sharing, while deeper forms of participation involve consultation, co-design, collaborative experimentation and partnership formation.

Different engagement levels require different forms of facilitation, stakeholder investment and governance coordination. Broad communication activities typically reach larger audiences with relatively limited interaction, whereas collaborative activities require smaller groups, stronger relationships and greater mutual commitment. The model therefore views stakeholder engagement as a progression pathway rather than as isolated communication activities.

Importantly, progression is not assumed to be automatic or uniform. Stakeholders may move between engagement levels according to changing priorities, institutional conditions, capacities and interests. The framework therefore supports adaptive and flexible participation pathways rather than fixed stakeholder trajectories ( IAP2 Federation, n.d.; Reed et al., 2018).

### 4.2 Co-creation over one-way dissemination

The second principle prioritises co-creation over one-way dissemination. Traditional dissemination approaches often focus on transferring project information to external audiences. In contrast, the proposed framework treats stakeholders as active contributors whose contextual knowledge, operational experience and practical perspectives shape innovation development.

Co-creation approaches enable stakeholders to participate in defining challenges, identifying priorities, shaping solution concepts and refining implementation pathways. Participatory methods such as stakeholder dialogues, ideation workshops, collaborative design exercises and experimentation environments support the integration of scientific, technical and local knowledge into the innovation process ( van Ewijk & Ros-Tonen, 2021; Gardezi et al., 2024).

This principle is particularly important in climate-smart agriculture and aquaculture contexts where technological solutions must respond to highly variable environmental, institutional and socio-economic conditions.

### 4.3 Contextual adaptation

The third principle is contextual adaptation. The framework recognises that innovation ecosystems differ significantly across regions, sectors, institutional environments and stakeholder groups. Consequently, stakeholder engagement methods cannot rely solely on standardised participation formats or universal implementation approaches.

The framework therefore emphasises adaptation to local governance conditions, cultural contexts, digital readiness levels, language environments, infrastructure conditions and stakeholder capacities. Engagement approaches may require substantial modification depending on regional realities, organisational maturity and ecosystem development stages.

Contextual adaptation is especially important in African digital agriculture and aquaculture ecosystems where environmental conditions, market structures, institutional capacities and digital infrastructures vary considerably across locations and actor groups ( FAO & ITU, 2022; African Union, 2024).

### 4.4 Multi-actor inclusivity

The fourth principle is multi-actor inclusivity. Climate-smart innovation ecosystems involve multiple actor categories including farmers, fishers, SMEs, researchers, innovation hubs, public authorities, development organisations, civil society actors and financial stakeholders. Each actor group contributes different forms of knowledge, legitimacy, resources and implementation capacity.

The framework therefore seeks to avoid narrow stakeholder representation or single-actor dominance. Instead, it promotes interaction between technical, institutional, policy, commercial and community perspectives. Multi-actor inclusivity supports cross-sector learning, ecosystem coordination and collaborative problem-solving by enabling stakeholders operating at different governance and operational levels to participate jointly in innovation processes ( EC: Multi-actor approach; World Bank, 2012).

The principle also recognises that stakeholder diversity strengthens innovation resilience and improves the likelihood that developed solutions will respond to real operational needs.

### 4.5 Iterative feedback and adaptive learning

The fifth principle is iterative feedback and adaptive learning. The framework assumes that innovation ecosystems evolve continuously through interaction, experimentation and stakeholder feedback. Engagement activities therefore function not only as communication channels but also as mechanisms for reflection, learning and adaptation.

Feedback loops may emerge through consultations, workshops, demonstrations, experimentation environments, governance dialogues and collaborative evaluation activities. These interactions allow priorities, implementation approaches and innovation pathways to evolve according to emerging insights and contextual changes.

Consequently, stakeholder engagement is understood as a continuous adaptive governance mechanism rather than a linear sequence of predefined communication activities ( Klerkx et al., 2010; Reed et al., 2018). This principle supports flexibility, responsiveness and continuous improvement throughout the innovation lifecycle.

### 4.6 Sustainability through collaborative ownership

The sixth principle focuses on sustainability through collaborative ownership. The framework assumes that long-term sustainability depends not only on technological functionality, but also on institutional embedding, stakeholder commitment, partnership continuity and local ownership.

As stakeholder engagement deepens, stakeholders increasingly contribute to governance, implementation continuity, network mobilisation and ecosystem support structures. Collaborative ownership may emerge through long-term partnerships, multi-stakeholder platforms, innovation communities, policy engagement and shared experimentation activities.

From this perspective, stakeholder engagement becomes directly connected to exploitation readiness, scalability and sustainability. The framework therefore links participation processes with broader ecosystem development and long-term innovation governance objectives ( Reed et al., 2018; World Bank, 2012).

The following chapter operationalises the methodological framework through empirical examples and stakeholder engagement activities implemented within the KijaniSpace project.

## 5. Operationalisation in KijaniSpace

The Multi-Actor Stakeholder Engagement Progression Model was operationalised in KijaniSpace through a combination of digital communication channels, stakeholder mapping, interviews, multi-actor workshops, ideation processes, innovation ecosystem activities and emerging collaboration structures across Kenya, Tanzania, Uganda and Europe. The operationalisation combined broad awareness-building activities with progressively deeper forms of participation, co-creation and collaboration. In practice, the project used four mutually supportive engagement strategies — **Inform, Consult, Involve and Collaborate** — to structure multi-actor participation and to connect communication, stakeholder engagement and uptake-oriented activities ( KijaniSpace: D6.2).

Importantly, the operationalisation did not follow a rigid linear sequence. Stakeholders entered the process through different pathways and engagement formats depending on their institutional role, technical capacity, geographic location, operational interests and innovation readiness. Some stakeholders participated only in awareness-oriented activities, while others progressed toward repeated workshop participation, ideation activities, pilot discussions and ecosystem-building processes. Consequently, the operationalisation functioned as an adaptive and iterative stakeholder progression system rather than as a fixed communication pipeline.

The engagement process combined broad outreach with progressively deepening participatory interaction. At the same time, stakeholder participation was monitored through stakeholder mapping and reporting, enabling the project to document the types of actors involved, their locations and their engagement formats. During the mid-term implementation phase of the project, more than 200 multi-actor stakeholders had been mapped, including technical users, farmer communities, policy actors and other innovation ecosystem actors ( KijaniSpace: D6.2).

### 5.1 Inform

The Inform stage focused on awareness-building, visibility and accessibility of project-related information. The objective was to reach broad stakeholder audiences across the Lake Victoria Basin and beyond through accessible digital and physical communication channels. The operationalisation combined website communication, newsletters, social media channels, videos, dissemination materials and participation in external events to create visibility around climate-smart agriculture, aquaculture and Space–IoT innovation activities.

The project website functioned as the main public information platform by providing project news, partner information, event visibility and public narratives related to the Space–IoT approach. Social media channels complemented the website by supporting professional networking, broader community outreach and audiovisual visibility of project activities ( KijaniSpace: D6.2).

During the mid-term implementation phase of the project, the Inform stage generated substantial outreach and visibility through digital communication channels, newsletters and public dissemination activities. These activities helped create initial awareness of the project and provided entry points for stakeholder participation within the broader innovation ecosystem ( KijaniSpace: D6.2).

Importantly, the Inform stage was not treated solely as a one-way dissemination activity. Instead, it functioned as the first layer of stakeholder progression by creating visibility, legitimacy and accessibility for subsequent consultation, co-creation and collaboration activities. In this sense, awareness-building supported the broader objective of gradually strengthening stakeholder interaction and ecosystem participation.

### 5.2 Consult

The Consult stage focused on generating contextual understanding of stakeholder needs, operational barriers and regional priorities related to climate-smart agriculture, aquaculture and digital innovation. Consultation activities included stakeholder interviews, needs assessments, dialogues, webinars and multi-actor discussions involving farmers, technical users, public authorities, researchers and innovation ecosystem actors.

The consultation process revealed that awareness and practical uptake of Earth Observation and IoT technologies remained limited across many stakeholder groups, despite strong interest in mobile-based advisory services, climate information and digital decision-support tools. Stakeholders also identified challenges related to fragmented institutional coordination, limited financing opportunities, uneven digital readiness and weak alignment between policy frameworks and local operational realities ( KijaniSpace: D2.1; D6.2).

Importantly, the consultation activities highlighted the need for strong contextual adaptation. Agricultural stakeholders emphasised issues such as soil fertility, climate variability, irrigation, water management and market access, while aquaculture stakeholders prioritised water quality monitoring, fish disease management, feeding systems and environmental risk management ( KijaniSpace: D2.1).

Rather than functioning only as information-gathering exercises, the consultation activities supported mutual learning between project actors and stakeholders. The process helped align technological possibilities with local realities, operational constraints and stakeholder expectations. In this sense, the Consult stage functioned as a bridge between broad awareness-building and deeper participatory involvement by strengthening contextual understanding, trust and relevance within the innovation ecosystem.

### 5.3 Involve

The Involve stage represented the transition from consultation toward active stakeholder participation in co-creation and innovation design. At this stage, stakeholders contributed directly to shaping use cases, identifying priorities, refining concepts and exploring implementation pathways.

KijaniSpace operationalised this stage through multi-actor workshops, ideation sessions, co-design activities and participatory prioritisation processes involving stakeholders from agriculture, aquaculture, research, governance, innovation support organisations and the private sector. These activities moved stakeholder engagement beyond information exchange toward collaborative problem-solving and shared concept development ( KijaniSpace: D2.2; D6.2).

The ideation workshops in Kenya and Tanzania played a particularly important role in operationalising participatory innovation. Using human-centred and design-thinking approaches, the workshops enabled stakeholders to jointly analyse challenges, identify operational priorities and explore potential Space–IoT solution pathways. The workshops brought together government representatives, researchers, innovators, SMEs, farmer organisations and community actors into a shared co-creation environment ( KijaniSpace: D2.2).

The co-creation process generated several converging priority themes for further development, including climate and early-warning advisory services, smart aquaculture support systems, soil and water monitoring, disease detection tools and market intelligence solutions. Importantly, the process also strengthened interaction between actor groups that would not typically collaborate within conventional dissemination-oriented project structures.

Rather than treating stakeholders as passive recipients of technological solutions, the Involve stage positioned them as active contributors to innovation development. In this sense, stakeholder engagement functioned simultaneously as a contextual learning process, a co-design mechanism and a collaborative innovation process supporting ownership and ecosystem learning.

### 5.4 Collaborate

The **Collaborate** stage focused on long-term ecosystem formation, partnership-building, governance interaction and sustainability-oriented collaboration. At this stage, stakeholders increasingly contribute not only to ideas and feedback, but also to ecosystem continuity, implementation pathways and future collaboration structures.

The Multi-Stakeholder Platform concept played a central role in this stage. The platform was designed to support knowledge exchange, accessible data repositories, AI- and metadata-supported data searchability, integration of traditional and indigenous knowledge with modern innovations, and collaboration among start-ups, SMEs, researchers and institutions ( KijaniSpace: D2.1).

The operationalisation identified the MSP as a flexible and context-sensitive mechanism based on a problem-driven framework, adaptive feedback, broad stakeholder representation, farmer participation, continuous feedback, peer learning and knowledge segmentation. It also emphasises farmer participation at three key stages: ideation, adoption and transition, supporting ownership and trust ( KijaniSpace: D2.1).

The collaborative governance dimension was further operationalised through proposed Technical Working Groups covering policy, research and innovation, and application. The operationalisation also highlights the need for broad representation from policymakers, researchers, farmers and NGOs, transparent decision-making and targeted capacity-building ( KijaniSpace: D2.1).

The Collaborate stage links stakeholder engagement directly with innovation governance, sustainability and exploitation readiness. Sustainability mechanisms identified in the project include institutional integration, capacity building, government and regional partnerships, monitoring and evaluation systems, knowledge repositories, long-term institutional and ecosystem support mechanisms ( KijaniSpace: D2.1).

Overall, the KijaniSpace operationalisation demonstrates that stakeholder engagement in Space–IoT innovation ecosystems can function simultaneously as a collaborative ecosystem-building and adaptive governance mechanism. The experience suggests that progressively deepening stakeholder engagement can support not only awareness and participation, but also collaborative ownership, ecosystem formation and long-term sustainability of innovation activities.

### 5.5 Synthesis of the operationalisation model

The operationalisation of the Multi-Actor Stakeholder Engagement Progression Model in KijaniSpace demonstrated how progressively deepening stakeholder engagement can support awareness-building, contextual learning, co-creation, ecosystem formation and collaborative ownership within Space–IoT innovation ecosystems.

Rather than functioning as isolated communication or dissemination activities, the engagement stages formed an interconnected and adaptive progression system in which stakeholders could move between different participation levels according to their interests, capacities, institutional roles and innovation readiness. The operationalisation also showed that different engagement stages generated different forms of value, ranging from broad visibility and awareness to co-design, partnership formation and sustainability-oriented collaboration.

Table 1 synthesises the main characteristics, operational mechanisms and expected outcomes associated with the four engagement stages.

**Table 1.** Synthesis of the multi-actor stakeholder engagement progression model operationalised in KijaniSpace.

Engagement stage	Main objective	Typical activities	Stakeholder role	Primary outcomes	Strategic value
Inform	Awareness and visibility	Website, social media, newsletters, videos, dissemination events	Information receiver	Visibility, awareness, initial interest	Entry point for engagement
Consult	Contextual understanding and needs identification	Interviews, surveys, dialogues, webinars, needs assessments	Knowledge contributor	Stakeholder insights, barriers, priorities	Trust-building and contextual adaptation
Involve	Co-creation and participatory innovation	Workshops, ideathons, co-design, prioritisation, roadmap development	Active co-creator	Shared concepts, pilot ideas, solution pathways	Ownership and ecosystem learning
Collaborate	Sustainability and ecosystem continuity	Multi-stakeholder platforms, partnerships, governance interaction, replication planning	Collaborative partner	Joint governance, networks, partnerships	Long-term sustainability and exploitation readiness

The table summarises the four stakeholder engagement stages — Inform, Consult, Involve and Collaborate — including their main objectives, typical activities, stakeholder roles, primary outcomes and strategic value within the operationalisation framework.

The operationalisation further demonstrated that stakeholder engagement intensity generally decreased as participation depth increased. Broad communication activities reached large audiences with relatively low interaction intensity, whereas collaborative activities involved fewer stakeholders but generated stronger trust, ownership and long-term ecosystem commitment. Consequently, the Multi-Actor Stakeholder Engagement Progression Model should not be interpreted as a linear funnel, but rather as an adaptive ecosystem in which stakeholders may simultaneously operate across multiple engagement levels.

## 6. Stakeholder progression and engagement dynamics

The operationalisation of the Multi-Actor Stakeholder Engagement Progression Model in KijaniSpace demonstrated that stakeholder engagement in innovation ecosystems is not static, linear or uniform. Instead, participation evolved through dynamic progression pathways in which stakeholders moved between different engagement levels according to their interests, capacities, institutional roles, contextual needs and innovation readiness ( Reed et al., 2018).

The KijaniSpace experience showed that stakeholder progression cannot be understood simply as movement through predefined communication stages. Rather, engagement deepened through repeated interaction, contextual learning, trust-building, co-creation and collaborative problem-solving. Stakeholders entered the ecosystem through multiple pathways and engagement formats, including website communication, social media interaction, webinars, interviews, workshops, ideation sessions and emerging collaboration structures ( KijaniSpace: D6.2). Some stakeholders remained primarily at awareness or consultation levels, while others progressed toward repeated participation, co-design activities and collaborative ecosystem engagement.

Importantly, the Multi-Actor Stakeholder Engagement Progression Model functioned as an adaptive ecosystem rather than as a one-directional participation funnel. Stakeholders could progress toward deeper participation, temporarily pause engagement, re-enter through different activities, or simultaneously participate across multiple engagement levels. This adaptive behaviour was particularly visible in the KijaniSpace ideation and workshop processes, where several actors participated repeatedly across consultation, involvement and collaboration-oriented activities ( KijaniSpace: D2.2).

### 6.1 Progression pathways

**Figure 1.**
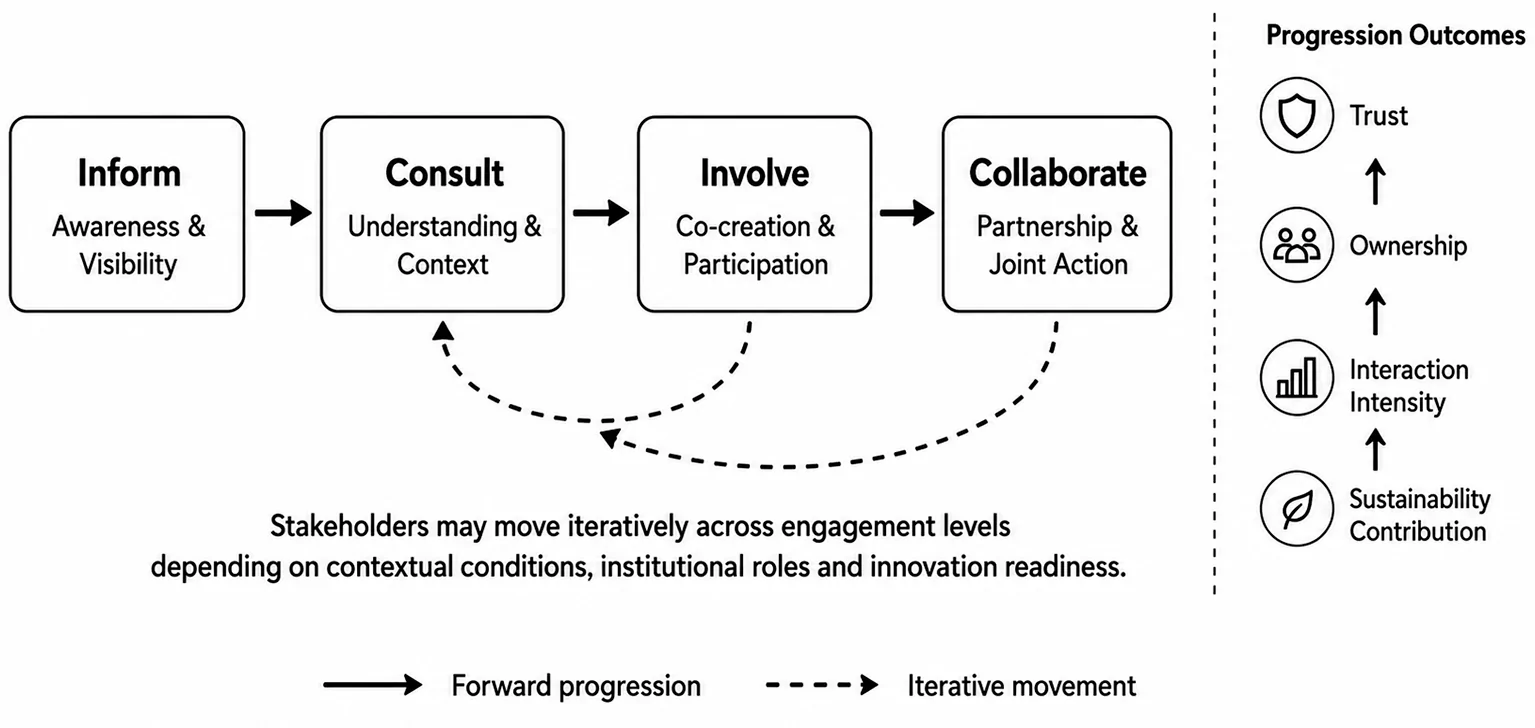
Adaptive and iterative stakeholder progression framework operationalised in the KijaniSpace context. The model illustrates dynamic movement across engagement levels and the progressive evolution of trust, ownership, interaction intensity and sustainability contribution. The figure illustrates the dynamic and non-linear progression of stakeholders across four interconnected engagement stages: Inform, Consult, Involve and Collaborate. The framework demonstrates how stakeholder interaction may progressively deepen through increasing levels of trust, ownership, participation intensity and sustainability contribution within Space–IoT innovation ecosystems. Stakeholders may move iteratively between engagement levels depending on contextual conditions, institutional roles and innovation readiness.

As illustrated in Figure 1, stakeholder progression functioned as an adaptive and iterative ecosystem process rather than as a fixed linear participation sequence. Stakeholders moved dynamically across engagement levels depending on contextual conditions, institutional roles, operational needs and innovation readiness. The KijaniSpace experience revealed several recurring stakeholder progression pathways within the innovation ecosystem.

One common pathway involved progression from awareness-oriented communication toward active co-creation. Stakeholders initially encountered the project through digital communication channels such as the project website, LinkedIn communication, newsletters or dissemination events. These awareness-oriented activities created visibility and initial recognition of Space–IoT opportunities in climate-smart agriculture and aquaculture contexts ( KijaniSpace: D6.2). Subsequent participation in webinars, interviews or stakeholder consultations then enabled stakeholders to deepen their understanding of the project and contribute contextual knowledge regarding operational needs, barriers and opportunities.

For some stakeholders, this consultation phase evolved further into active participation in ideation workshops and co-design activities. In these cases, stakeholders transitioned from information recipients and knowledge contributors toward active co-creators participating in challenge definition, prioritisation, solution development and roadmap discussions. The ideation workshops in Kenya and Tanzania demonstrated how participatory methodologies can progressively deepen stakeholder interaction by moving participants from problem identification toward collaborative concept development and implementation-oriented thinking ( KijaniSpace: D2.2).

Another progression pathway emerged through institutional and ecosystem collaboration. Public authorities, innovation hubs, researchers and SMEs initially engaged through consultation and workshop participation, but gradually moved toward discussions related to governance structures, partnership formation, ecosystem continuity, pilot implementation, replication opportunities and sustainability-oriented collaboration. This progression pathway became particularly visible in discussions surrounding the Multi-Stakeholder Platform concept and regional ecosystem coordination mechanisms.

The operationalisation also demonstrated that progression intensity varied considerably between stakeholders. Broad awareness-oriented activities reached large audiences with relatively low interaction intensity, whereas collaborative activities involved fewer actors but generated stronger trust, ownership and long-term engagement potential. Consequently, the framework should not be interpreted as a hierarchical filtering mechanism, but as a flexible ecosystem of overlapping participation opportunities.

### 6.2 Trust-building mechanisms

Trust-building emerged as a central factor influencing stakeholder progression within the KijaniSpace innovation ecosystem. Previous participation research has shown that trust strongly affects the continuity, quality and effectiveness of stakeholder engagement processes ( Reed, 2008; Reed et al., 2018). The KijaniSpace experience similarly demonstrated that stakeholders were more willing to deepen participation when engagement activities were perceived as relevant, inclusive and responsive to local realities.

Trust developed progressively through repeated interaction, participatory dialogue and collaborative problem-solving. Consultation activities, workshops and co-creation sessions enabled stakeholders to move beyond passive information reception toward more active contribution and interaction. This gradual deepening of participation helped strengthen familiarity between stakeholders and project actors while reducing perceived distance between technological concepts and operational realities.

Contextual relevance played a particularly important role in trust formation. Stakeholders responded more positively when discussions addressed practical challenges such as climate variability, water management, fish health, soil fertility and market-related constraints within their own operational environments ( KijaniSpace: D2.1; D2.2). Participatory methodologies, including ideation workshops and collaborative prioritisation exercises, further strengthened trust by enabling stakeholders to influence discussions and contribute to shaping innovation pathways.

The operationalisation also suggested that trust-building was cumulative rather than immediate. Stakeholders often progressed toward deeper engagement only after repeated exposure to communication activities, consultation processes and collaborative interaction. Consequently, trust emerged not as a separate activity, but as an evolving relational outcome generated through continuous participation, contextual adaptation and adaptive ecosystem interaction.

### 6.3 Emergence of co-ownership

The KijaniSpace operationalisation further demonstrated that stakeholder engagement can progressively evolve toward forms of collaborative ownership. Co-ownership did not emerge through formal transfer of responsibilities alone, but through increasing stakeholder participation in defining priorities, shaping use cases, contributing contextual knowledge and discussing long-term implementation pathways.

In the ideation workshops, stakeholders actively participated in identifying climate-smart use cases, defining operational priorities and discussing implementation opportunities for Space–IoT solutions ( KijaniSpace: D2.2). These activities contributed to a transition from externally presented technological concepts toward jointly discussed and locally contextualised innovation opportunities.

The emergence of co-ownership was particularly visible in discussions related to Multi-Stakeholder Platform structures, regional ecosystem coordination, local innovation ecosystems, startup participation and future collaboration opportunities. The operationalisation emphasised the importance of broad representation, adaptive governance, farmer participation and collaborative support systems for long-term sustainability of the innovation ecosystem ( KijaniSpace: D2.1).

The process also demonstrated that collaborative ownership depends strongly on whether stakeholders perceive innovation activities as locally relevant, adaptable, inclusive and connected to long-term ecosystem benefits. Similar observations have been made in Agricultural Innovation Systems literature, which highlights that sustainable innovation ecosystems depend on interaction, institutional support and shared learning rather than technology transfer alone ( World Bank, 2012; Klerkx et al., 2010).

Consequently, co-ownership should be understood not as a single engagement outcome, but as an evolving relational process emerging through repeated participation and ecosystem interaction.

### 6.4 Feedback loops and adaptive governance

The KijaniSpace operationalisation highlighted the importance of feedback loops and adaptive governance within stakeholder progression dynamics. Stakeholder interaction continuously influenced the evolution of priorities, engagement activities and innovation pathways throughout the operational process.

Consultation activities generated contextual insights that informed workshop themes, ideation priorities and ecosystem discussions. In turn, co-creation activities refined understanding of stakeholder needs, implementation barriers and collaboration opportunities, which subsequently influenced communication narratives, sustainability considerations and emerging governance structures ( KijaniSpace: D2.1; D2.2; D6.2).

Rather than functioning as a predefined communication sequence, the engagement process operated as a continuous adaptive learning system in which stakeholder interaction supported knowledge generation, collective learning and iterative adjustment of activities. This aligns with adaptive innovation governance literature emphasising continuous interaction, institutional learning and responsiveness within complex innovation ecosystems ( Klerkx et al., 2010).

The operationalisation further demonstrated that adaptive governance becomes increasingly important in environments characterised by technological uncertainty, institutional diversity and varying levels of digital readiness. Consequently, the framework supports flexible and context-sensitive participation pathways rather than rigid stakeholder structures or fixed implementation sequences.

### 6.5 Synthesis of stakeholder progression dynamics

The KijaniSpace experience demonstrated that stakeholder engagement in Space–IoT innovation ecosystems functions as a dynamic and adaptive progression process rather than as a linear communication sequence. Stakeholders moved between different engagement levels through repeated interaction, contextual learning, co-creation and collaborative governance activities.

Different engagement stages generated different forms of value. Awareness-oriented communication increased visibility and ecosystem entry points, consultation activities generated contextual understanding, co-creation strengthened participatory innovation and collaborative activities supported partnership formation, ecosystem continuity and sustainability-oriented governance.

The operationalisation further highlighted the importance of trust-building, contextual adaptation and iterative feedback loops. Stakeholders were more willing to deepen participation when engagement activities reflected local realities, enabled meaningful contribution and responded to stakeholder needs.

Importantly, stakeholder engagement functioned not only as a dissemination mechanism, but also as a learning, co-creation and adaptive governance mechanism. The findings suggest that progressively deepening stakeholder engagement can support ecosystem formation, collaborative ownership and long-term sustainability of innovation activities in complex multi-actor environments.

## 7. Implications for sustainability and exploitation

The KijaniSpace operationalisation demonstrated that progressively deepening stakeholder engagement can strengthen sustainability and exploitation readiness within Space–IoT innovation ecosystems. While the Inform and Consult stages supported visibility, contextual understanding and identification of operational barriers, deeper sustainability potential emerged primarily during the Involve and Collaborate stages, where stakeholders participated in co-creation, ecosystem discussions, governance interaction and implementation-oriented dialogue. These activities strengthened trust, institutional alignment, contextual relevance and collaborative ownership around the innovation process.

The findings suggest that sustainability in Space–IoT innovation ecosystems should not be understood solely through technological deployment or commercialisation pathways. Instead, sustainability may emerge through multiple interconnected mechanisms, including institutional embedding, capacity-building, ecosystem coordination, local innovation support structures and collaborative governance processes. In the KijaniSpace context, stakeholder interaction contributed not only to communication and participation, but also to ecosystem formation and continuity-oriented thinking.

The operationalisation further demonstrated that co-creation can strengthen exploitation readiness by reducing the gap between technological concepts and operational realities. Stakeholders participating in workshops, consultations and collaborative discussions contributed contextual knowledge, identified implementation barriers and helped refine locally relevant application pathways. This process strengthened the practical relevance and legitimacy of the innovation activities while increasing stakeholder familiarity with the developed approaches.

The role of innovation intermediaries, SMEs, innovation hubs and regional organisations also appeared important for long-term sustainability. These actors can potentially support continuation, adaptation and future uptake of digital applications beyond project-based activities. Consequently, stakeholder engagement functioned not only as a dissemination mechanism, but also as an ecosystem activation mechanism connecting technical development, local innovation environments and institutional support structures.

The KijaniSpace experience also highlighted the importance of institutional interaction. Long-term continuity may depend partly on whether public authorities, regional organisations, educational institutions and governance actors perceive the developed approaches as compatible with broader regional development priorities and climate-smart transition objectives. Stakeholder engagement therefore contributed not only to technological experimentation, but also to policy relevance, institutional learning and governance dialogue.

Importantly, the operationalisation suggested that sustainability develops progressively through interaction intensity. Broad communication activities supported visibility and ecosystem entry points, whereas deeper collaborative interaction generated stronger ownership, partnership continuity and long-term commitment. In this sense, stakeholder progression simultaneously supported communication, learning, co-creation and sustainability-oriented ecosystem development.

At the same time, the operationalisation highlighted several challenges relevant to sustainability and exploitation. Innovation ecosystems may face uneven stakeholder capacities, limited institutional resources, varying digital readiness and fragmented governance environments. These factors may constrain long-term continuity even when stakeholder engagement processes are active and participatory. Consequently, sustainability requires continuous ecosystem-building, iterative adaptation and realistic continuation pathways rather than reliance on isolated dissemination or pilot activities alone.

Overall, the findings suggest that sustainability and exploitation readiness in Space–IoT innovation ecosystems depend strongly on the depth and quality of stakeholder engagement. Progressively deepening participation can strengthen collaborative ownership, institutional embedding and ecosystem continuity, thereby supporting more resilient and adaptive innovation processes in complex multi-actor environments.

## 8. Transferability and replicability

The KijaniSpace experience suggests that the Multi-Actor Stakeholder Engagement Progression Model may have broader applicability beyond the specific Lake Victoria Basin context. Although operationalised within a Space–IoT innovation ecosystem focused on climate-smart agriculture and aquaculture, the framework addresses several challenges that are common across Horizon Europe projects and multi-actor innovation initiatives more generally. These include fragmented stakeholder ecosystems, weak long-term ownership, limited institutional embedding and the tendency to treat stakeholder engagement primarily as dissemination rather than as an adaptive governance mechanism.

One important aspect of transferability is that the framework is not technology-specific. While KijaniSpace focused on Copernicus Earth Observation, IoT integration and climate-smart applications, the progression logic itself can potentially support a much wider range of innovation ecosystems involving digital transformation, sustainability transitions, regional development or participatory governance. The four interconnected engagement stages — Inform, Consult, Involve and Collaborate — provide a flexible structure that can be adapted to different technological, institutional and geographical contexts.

The framework may be particularly relevant for Horizon Europe and international cooperation projects operating in complex multi-actor environments. Many research and innovation projects successfully disseminate outputs and organise stakeholder events, but fewer operationalise systematic progression from communication toward co-creation, collaborative ownership and sustainability-oriented interaction. The KijaniSpace operationalisation demonstrates how stakeholder engagement can evolve from awareness-building toward ecosystem formation, institutional dialogue and long-term collaboration.

The findings may also be relevant for AU–EU innovation ecosystems where technological innovation depends strongly on contextual adaptation, local ownership and institutional coordination. The operationalisation highlighted the importance of trust-building, participatory interaction and responsiveness to local realities, particularly in environments characterised by varying digital readiness, institutional diversity and resource constraints. These observations align with broader digital agriculture and Agricultural Innovation Systems literature emphasising that innovation uptake depends not only on technology availability, but also on interaction, facilitation, governance support and ecosystem learning ( Klerkx et al., 2010; World Bank, 2012).

The transferability potential is especially visible in climate-smart agriculture and digital innovation ecosystems where solutions must operate across multiple actor groups, governance levels and implementation environments. The framework supports this complexity by combining broad communication activities with progressively deeper forms of participation and adaptive governance. Because stakeholders may simultaneously operate across multiple engagement levels, the model avoids overly rigid participation structures and instead supports flexible ecosystem interaction.

The KijaniSpace experience also suggests that the framework may support regional innovation systems and living lab-type environments where long-term sustainability depends on continuous stakeholder interaction rather than isolated pilot activities. The progression logic can help connect communication, consultation, co-design, experimentation and collaborative governance into a more integrated ecosystem process.

At the same time, replicability should not be understood as direct copying of operational activities. The framework itself is transferable, but operationalisation requires contextual adaptation. Engagement methods, communication channels, institutional arrangements and participation formats may need substantial modification depending on governance conditions, cultural contexts, technological maturity, language environments and stakeholder capacities. Consequently, replicability depends less on reproducing individual activities and more on applying the underlying progression principles in locally relevant ways.

Overall, the KijaniSpace operationalisation suggests that progressively deepening stakeholder engagement may provide a transferable methodological approach for strengthening participation, collaborative ownership and sustainability in complex innovation ecosystems operating across institutional, technological and regional boundaries.

## 9. Limitations

### 9.1 Evolving project context

This study was conducted during the ongoing implementation phase of the KijaniSpace project. Consequently, several innovation activities, pilot demonstrations, ecosystem-building processes and sustainability pathways were still evolving at the time of writing. The article therefore focuses primarily on methodological operationalisation and stakeholder progression dynamics rather than on long-term impact assessment or final exploitation outcomes. Some collaborative structures, continuation mechanisms and institutional embedding processes may continue to develop beyond the current observation period.

### 9.2 Limited long-term impact evidence

Although the operationalisation generated evidence regarding stakeholder interaction, participation dynamics and co-creation processes, the long-term impacts of the framework remain only partially measurable. At this stage, it is not yet possible to comprehensively assess sustained behavioural change, long-term technology adoption, institutional continuity or long-term exploitation outcomes.

The findings should therefore be interpreted primarily as evidence of stakeholder engagement progression and adaptive governance processes rather than definitive proof of long-term sustainability impacts.

### 9.3 Context-specific operationalisation

The framework was operationalised within a specific regional and institutional context centred on the Lake Victoria Basin in East Africa. The operational environment included particular combinations of climate-smart agriculture, aquaculture, Space–IoT technologies, regional governance structures, and AU–EU collaboration dynamics.

Although the Multi-Actor Stakeholder Engagement Progression Model itself may have broader applicability, operationalisation approaches are likely to require contextual adaptation in other geographical, institutional and technological environments.

### 9.4 Diversity of African innovation contexts

The article does not assume that African innovation ecosystems form a homogeneous operational environment. Substantial differences exist across regions regarding digital infrastructure, institutional capacity, innovation support ecosystems, language environments, governance structures, and stakeholder readiness.

Consequently, the operationalisation observed in KijaniSpace should not be interpreted as universally representative of African digital agriculture contexts. Replication and transferability require sensitivity to local socio-economic, cultural and institutional conditions.

### 9.5 Participation and representation limitations

Like many participatory innovation processes, the operationalisation may also have been influenced by unequal participation intensity, differing stakeholder capacities and varying levels of digital accessibility. Some stakeholder groups may have participated more actively than others due to institutional resources, technical expertise, connectivity or organisational positioning. Although the framework sought broad multi-actor inclusivity, fully balanced participation across all actor categories remains difficult to achieve in complex innovation ecosystems.

## 10. Conclusions

### 10.1 Main contribution of the article

This article proposed and operationalised a Multi-Actor Stakeholder Engagement Progression Model for Space–IoT innovation ecosystems in climate-smart agriculture and aquaculture contexts. The study demonstrated that stakeholder engagement can be understood not merely as dissemination or communication support, but as a dynamic innovation governance mechanism connecting awareness-building, consultation, co-creation, collaborative ownership and sustainability-oriented ecosystem development.

The proposed framework combined insights from participatory innovation, Agricultural Innovation Systems, living lab approaches and adaptive governance literature into an operational Multi-Actor Stakeholder Engagement Progression Model structured around four interconnected engagement stages: Inform, Consult, Involve and Collaborate. Importantly, the study showed that stakeholder progression is not linear, but adaptive and iterative. Stakeholders may deepen, pause, re-enter or simultaneously participate across multiple engagement levels depending on contextual conditions, institutional maturity and innovation readiness.

### 10.2 Main findings from KijaniSpace

The operationalisation within the KijaniSpace project demonstrated that progressively deepening stakeholder engagement can strengthen trust, contextual learning and collaborative ecosystem participation within complex digital innovation ecosystems. Broad communication activities supported awareness and visibility, while consultation activities generated contextual understanding of operational realities. Co-creation activities strengthened interaction intensity, collaborative learning and locally contextualised solution development, whereas collaboration-oriented activities supported ecosystem formation, institutional dialogue and sustainability-oriented interaction.

The findings also highlighted the importance of repeated interaction, contextual adaptation and participatory methodologies. Stakeholders were more willing to deepen participation when engagement activities reflected local operational realities, enabled meaningful contribution and supported collaborative problem-solving. Consequently, stakeholder engagement functioned as an adaptive governance and collaborative learning mechanism within the innovation ecosystem.

The KijaniSpace experience further demonstrated that sustainability and exploitation readiness depend strongly on engagement depth. Awareness alone was insufficient to generate ownership or long-term ecosystem continuity. More sustainable engagement dynamics emerged when stakeholders progressively participated in co-design, collaborative discussions, ecosystem coordination and governance-oriented interaction.

### 10.3 Methodological and practical implications

The article contributes to ongoing discussions regarding the role of stakeholder engagement in Horizon Europe and international innovation projects. Many research and innovation initiatives disseminate outputs and organise stakeholder events, but fewer operationalise systematic progression from communication toward collaborative ownership and sustainability-oriented participation. The proposed framework therefore provides a practical methodological approach for connecting stakeholder engagement with co-creation, ecosystem governance and long-term sustainability.

The findings may be particularly relevant for AU–EU cooperation projects and digital agriculture ecosystems operating in complex institutional and socio-technical environments. The operationalisation demonstrated that innovation uptake depends not only on technological functionality, but also on trust-building, contextual adaptation, institutional interaction and collaborative ecosystem formation.

More broadly, the framework suggests that stakeholder engagement should be integrated into innovation governance from the beginning of a project rather than treated as a separate dissemination layer implemented alongside technical development activities. Progressively deepening participation can strengthen ecosystem resilience, collaborative ownership and long-term continuation potential in complex multi-actor innovation environments.

### 10.4 Future research and development needs

Because the study was conducted during the ongoing implementation phase of the KijaniSpace project, long-term impacts related to institutional continuity, technology adoption and exploitation outcomes remain only partially observable. Future research could therefore examine how progression-based stakeholder engagement influences long-term sustainability, ecosystem continuity and practical uptake beyond project implementation periods.

Comparative studies across different geographical and technological contexts would also help evaluate the transferability of the framework. Additional research could further explore quantitative participation analytics, stakeholder network dynamics and long-term governance evolution within digital innovation ecosystems.

Overall, the KijaniSpace experience suggests that progressively deepening stakeholder engagement may provide an important pathway toward more inclusive, adaptive and sustainable innovation ecosystems in climate-smart agriculture and digital transformation contexts.

### 10.5 Final conclusion

Stakeholder engagement should not be treated as a separate dissemination activity conducted around innovation ecosystems. Instead, it should be understood as a central adaptive governance mechanism through which innovation ecosystems are co-created, sustained and embedded into long-term institutional and societal structures.

## Ethics and consent

The study is based primarily on analysis of public project materials, stakeholder engagement activities, workshop processes and communication activities conducted within the KijaniSpace project framework.

The operational activities described in the article involved participatory workshops, stakeholder consultations, webinars, interviews and multi-actor engagement processes related to climate-smart agriculture, aquaculture and digital innovation ecosystems. Participation in these activities was voluntary and based on informed participation within the normal operational context of the project.

The article does not publish personal stakeholder-level information, sensitive personal data or identifiable interview content. The analysis focuses on aggregated operational observations, methodological processes and stakeholder engagement dynamics rather than on individual participants.

Communication and stakeholder engagement activities were conducted in accordance with applicable ethical and data protection principles, including GDPR-compliant communication and consent practices where relevant. Public communication analytics and participation figures used in the article are presented only in aggregated form.

Because the article does not involve medical research, clinical trials or the publication of identifiable personal data, no separate formal ethical approval process was required for this methodological and operational analysis.

## Use of generative AI tools

Generative AI tools were used during the preparation of the paper to support language refinement, structural editing, formatting suggestions and drafting assistance. Specifically, ChatGPT (OpenAI GPT-5 series) was used interactively to support text development, condensation, consistency checking and editorial refinement. All scientific interpretation, methodological framing, operational descriptions, conclusions and final content decisions were reviewed, validated and approved by the authors. The authors take full responsibility for the accuracy, originality and integrity of the paper.

## Data Availability

This study is based primarily on publicly available project materials, methodological documentation, communication analytics and stakeholder engagement reports produced within the KijaniSpace project framework. Publicly available sources used in the study include the KijaniSpace project website, public communication materials and social media communication channels, and public deliverables available through the European Commission CORDIS platform. The study did not generate or publish personal stakeholder-level datasets. Some operational stakeholder engagement materials, workshop interaction records and internal ecosystem-building discussions contain potentially sensitive organisational or personal information and are therefore not publicly shared. Aggregated communication metrics, workshop participation figures and operational descriptions used in the article are included within the paper and referenced public project materials.
