# Retrospective Analysis of Stakeholder Engagement Activities in a Horizon Europe Multi-Actor Project: Evidence from KijaniSpace

**DOI:** 10.12688/openreseurope.23950.2

**Published:** 2026-08-21

**Authors:** Tuula-Maija Löytty

**Affiliations:** 1 Smart & Lean Hub Oy, Lahti, 15320, Finland

**Keywords:** stakeholder engagement, adaptive governance, participatory innovation, co-creation, Agricultural Innovation Systems, living labs, digital agriculture, climate-smart agriculture, innovation ecosystems, sustainability, multi-actor approach, Space–IoT integration

## Abstract

**Background:**

Stakeholder engagement is increasingly recognised as an important component of digital agriculture and climate-smart innovation projects. However, communication, consultation, co-design and collaboration activities are often implemented separately, without a common analytical structure for examining how they relate during project implementation.

**Case study:**

This article presents a retrospective descriptive case study of stakeholder engagement in KijaniSpace, a Horizon Europe project using Copernicus Earth Observation and Internet of Things technologies to support climate-smart agriculture and aquaculture in the Lake Victoria Basin. Stakeholder engagement activities were planned as operational project tasks within the Horizon Europe multi-actor approach but were not originally conceptualised as components of a unified framework.

**Methods:**

The study analysed stakeholder mapping datasets, project deliverables, workshop and ideation reports, and communication and dissemination records. Activities were classified according to their primary objective, interaction format and intended stakeholder role. Drawing on the IAP2 Spectrum of Public Participation, Agricultural Innovation Systems, living lab research and multi-actor innovation literature, the activities were organised into four analytical categories: Inform, Consult, Involve and Collaborate.

**Results:**

Activities corresponding to all four categories were identified. Inform activities supported awareness and access to project information. Consult activities generated contextual knowledge on stakeholder needs, barriers and regional priorities. Involve activities created opportunities for problem definition, ideation, co-design and participatory prioritisation. Collaborate activities addressed partnership development, governance dialogue, ecosystem coordination and continuity planning. The categories overlapped, and the evidence did not demonstrate sequential progression of individual stakeholders.

**Conclusions:**

The KijaniSpace case shows how heterogeneous stakeholder engagement activities can be retrospectively organised through a common analytical framework. The framework is an interpretive classification structure rather than a validated methodology or measured progression model. It may support more transparent documentation and comparative analysis, while effects on trust, ownership, institutional embedding and long-term sustainability require longitudinal and participant-based research.

## 1. Introduction

Climate-smart agriculture and digital innovation projects increasingly depend on collaboration among researchers, technology developers, public authorities, SMEs, innovation intermediaries and end-users. In such environments, stakeholder engagement extends beyond communication and dissemination to include consultation, co-creation, collaborative learning and the development of partnerships that support innovation uptake and long-term sustainability ( Reed, 2008; World Bank, 2012). This is particularly important in projects that combine emerging digital technologies, environmental monitoring and local development objectives across diverse institutional, socio-economic and geographical contexts.

Within Horizon Europe, stakeholder engagement is embedded in the broader **multi-actor approach**, which promotes the active involvement of researchers, practitioners, businesses, public authorities, civil society organisations and other relevant actors throughout research and innovation processes. Rather than treating stakeholders as passive recipients of research outputs, the multi-actor approach encourages continuous interaction, knowledge exchange and co-creation from project design through implementation, demonstration and exploitation. Consequently, Horizon Europe projects commonly include communication, consultation, participatory workshops, stakeholder dialogues and collaborative activities as integral operational components of project implementation rather than as stand-alone dissemination tasks.

These considerations are particularly relevant in AU–EU innovation contexts, where digital solutions must operate across diverse governance systems, varying levels of digital readiness and highly context-specific agricultural and environmental conditions. In climate-smart agriculture and aquaculture, the uptake of Earth Observation, Internet of Things (IoT) and digital advisory solutions depends not only on technological performance but also on contextual adaptation, institutional coordination, stakeholder participation and collaborative learning ( FAO & ITU, 2022; African Union, 2024). Agricultural Innovation Systems research similarly emphasises that innovation emerges through interaction, facilitation, knowledge exchange and institutional collaboration rather than through linear technology transfer alone ( Klerkx et al., 2010; van Ewijk & Ros-Tonen, 2021).

Participatory innovation, living lab approaches and stakeholder participation frameworks provide valuable conceptual perspectives for understanding how different forms of engagement contribute to innovation processes ( Reed et al., 2018; Gardezi et al., 2024; Toffolini et al., 2023). However, these approaches primarily describe principles of stakeholder participation rather than how stakeholder engagement is operationalised and evolves within the implementation of large multi-partner research and innovation projects. There remains a need for case-based studies that examine how stakeholder engagement activities implemented within Horizon Europe projects can be interpreted, organised and analysed in relation to established participation theory.

This article addresses that need through a descriptive case study of the **KijaniSpace** project, a Horizon Europe innovation project supporting climate-smart agriculture and aquaculture in the Lake Victoria Basin through the integration of Copernicus Earth Observation, Internet of Things technologies and digital innovation. KijaniSpace was conceived from the outset as a multi-actor project. The Grant Agreement incorporated a broad range of stakeholder engagement activities, including communication and dissemination, stakeholder mapping, needs assessments, interviews, consultations, workshops, co-design activities, ecosystem-building initiatives and collaboration-oriented mechanisms. These activities were designed as operational project tasks supporting implementation and innovation uptake rather than as components of an explicit conceptual stakeholder engagement framework.

Although stakeholder engagement formed an integral part of project implementation, the relationships among the different engagement activities were not originally articulated through a common analytical structure. Communication, consultation, co-creation and collaboration activities were planned and implemented within different work packages to support project objectives, but they were not conceptualised as elements of a unified stakeholder engagement framework. This provided an opportunity for retrospective analysis of the implemented activities and their relationships.

Accordingly, this paper retrospectively analyses documented stakeholder engagement activities implemented within KijaniSpace and organises them using concepts derived from established stakeholder participation literature, including the IAP2 Spectrum of Public Participation, Agricultural Innovation Systems, living lab approaches and multi-actor innovation research. Based on this retrospective interpretation, the paper presents the **Multi-Actor Stakeholder Engagement Progression Framework** as an analytical framework for organising and interpreting documented engagement activities. The framework was developed after implementation to conceptualise project practice rather than to prescribe stakeholder engagement methods or validate a new methodology.

Using project documentation, including communication activities, stakeholder mapping, interviews, consultations, workshops, ideation sessions, co-design activities and emerging collaboration structures, the paper examines how stakeholder engagement was organised during project implementation and how different forms of participation contributed to contextual learning, co-creation and collaboration-oriented planning. The framework classifies documented activities into four analytical engagement categories— **Inform, Consult, Involve** and **Collaborate**—that represent conceptually different forms of stakeholder interaction. These categories are not treated as fixed or sequential stages through which all stakeholders progress. Rather, they provide an analytical structure for interpreting documented engagement activities that may overlap, recur or occur independently depending on stakeholder roles, project objectives and contextual circumstances.

The analysis does not seek to demonstrate causal relationships between stakeholder engagement and outcomes such as trust, ownership, institutional embedding, exploitation readiness or long-term sustainability. These outcomes were not directly measured within the project and are therefore discussed only as possible interpretations informed by the literature and the documented implementation experience.

The study addresses the following research question:

**How can stakeholder engagement activities implemented within a Horizon Europe multi-actor project be retrospectively organised and interpreted using an analytical stakeholder engagement framework, and what insights does this provide into communication, consultation, co-creation and collaboration during project implementation?**

The contribution of this article is not the development of a new stakeholder engagement methodology. Rather, it demonstrates how stakeholder engagement activities implemented within a Horizon Europe multi-actor project can be retrospectively conceptualised into a coherent analytical framework informed by established stakeholder participation theory. The framework provides a structured way to interpret relationships among communication, consultation, involvement and collaboration within the KijaniSpace case and offers an analytical approach that may support comparative analysis of stakeholder engagement in future multi-actor research and innovation projects.

## 2. Background and conceptual context

Stakeholder engagement has become an increasingly important component of research and innovation projects addressing complex societal challenges such as climate-smart agriculture, digital transformation and environmental sustainability. Contemporary innovation policy recognises that technological advances alone are often insufficient to achieve lasting impact. Instead, successful innovation depends on continuous interaction among researchers, technology developers, businesses, public authorities, intermediaries and end-users throughout the innovation process ( Reed, 2008; World Bank, 2012).

Within Horizon Europe, this perspective is reflected in the **multi-actor approach**, which promotes collaboration among diverse stakeholder groups from project conception through implementation, demonstration and exploitation. Rather than viewing communication and dissemination as isolated project activities, the multi-actor approach encourages continuous knowledge exchange, co-creation and shared learning so that innovations respond to practical needs and operating environments ( European Commission, Multi-Actor Approach; EU CAP Network, 2017).

Within this article, **digital agriculture** refers to the application of digital technologies, including Earth Observation, Internet of Things (IoT), sensors, artificial intelligence and digital advisory services, to support agricultural decision-making and resource management. **Climate-smart agriculture** refers to approaches that seek simultaneously to improve productivity, strengthen resilience to climate change and, where appropriate, reduce environmental impacts. In KijaniSpace, Space–IoT technologies are considered enabling technologies that support climate-smart agriculture by providing information and digital services for more informed decision-making.

Several complementary bodies of literature informed the retrospective interpretation presented in this study.

Agricultural Innovation Systems (AIS) research emphasises that innovation emerges through interaction among multiple actors rather than through linear technology transfer. Innovation depends on knowledge exchange, institutional coordination, facilitation and collaborative learning involving research organisations, public authorities, businesses, intermediaries and end-users ( World Bank, 2012; Klerkx et al., 2010). This perspective highlights the importance of stakeholder interaction throughout innovation processes rather than only during dissemination.

Participatory innovation and living lab research similarly emphasise the active involvement of stakeholders in identifying needs, defining problems, co-designing solutions and evaluating innovations under real-world conditions ( Gardezi et al., 2024; Toffolini et al., 2023). These approaches recognise that stakeholders contribute valuable contextual, experiential and operational knowledge that complements scientific and technical expertise. Rather than treating participation as a single event, they describe engagement as an iterative process in which learning and adaptation occur throughout project implementation.

The literature on stakeholder participation provides additional conceptual perspectives for understanding different forms of engagement. The IAP2 Spectrum of Public Participation distinguishes among increasing forms of participation, ranging from informing and consulting to involving, collaborating and empowering stakeholders ( IAP2 Federation, n.d.). Although originally developed for public participation, the spectrum provides a useful conceptual reference for distinguishing different forms of stakeholder interaction. Reed (2008) and Reed et al. (2018) further emphasise that effective stakeholder engagement depends on trust, legitimacy, contextual adaptation and the quality of interaction rather than on the application of standard participation procedures.

Research on digital agriculture in Sub-Saharan Africa further highlights the importance of contextual adaptation. FAO and ITU (2022) identify digital infrastructure, institutional capacity, governance arrangements and digital literacy as important factors influencing digital transformation. Similarly, the African Union Digital Agriculture Strategy ( African Union, 2024) emphasises local ownership, entrepreneurship, ecosystem development and collaborative innovation as prerequisites for sustainable digital transformation. These studies suggest that stakeholder engagement should be adapted to local institutional, socio-economic and environmental conditions rather than implemented through uniform participation models.

Taken together, these complementary perspectives provide a conceptual lens for interpreting stakeholder engagement within the KijaniSpace project. Rather than serving as a blueprint for project implementation, they offer theoretical concepts that help explain how different stakeholder engagement activities contributed to communication, contextual understanding, co-creation and collaboration during the implementation of a complex multi-actor innovation project.

Accordingly, this study does not seek to compare or validate existing stakeholder participation frameworks. Instead, these established theoretical perspectives are used retrospectively to organise and interpret the stakeholder engagement activities documented during the implementation of KijaniSpace. The analytical framework presented in the following chapter synthesises these concepts into a coherent structure for examining how different forms of stakeholder engagement were represented within the project and how they related to the broader objectives of collaborative innovation, contextual learning and ecosystem development.

## 3. KijaniSpace context

KijaniSpace is a Horizon Europe research and innovation project that examines how Copernicus Earth Observation services and Internet of Things (IoT) technologies can support climate-smart agriculture and aquaculture in the Lake Victoria Basin of East Africa. The project operates across Kenya, Tanzania and Uganda and brings together European and African partners representing research organisations, SMEs, innovation hubs, regional organisations and technical experts. Its overall objective is to develop, demonstrate and promote digital services that improve decision-making, environmental monitoring and sustainable management of agricultural and aquaculture systems ( KijaniSpace project website, n.d.; KijaniSpace Grant Agreement, 2024).

The Lake Victoria Basin is a strategically important agricultural, fisheries and aquaculture region that supports the livelihoods of millions of people. At the same time, the region faces challenges associated with climate variability, environmental degradation, declining fish stocks, water quality and access to timely information for decision-making ( Lake Victoria Basin Commission, n.d.; FAO, 2022; IPCC, 2022). KijaniSpace project documentation similarly identifies climate variability, water-quality management, soil and crop health, fish diseases, market access and fragmented information systems as important regional challenges.

To address these challenges, KijaniSpace combines Copernicus Earth Observation data, IoT technologies and digital innovation methods to develop applications supporting crop production, aquaculture, water management and environmental monitoring. A central technical concept is the KijaniBox, also referred to as the Space–IoT Box, which provides an integration environment for combining space-based data, sensor technologies and user-oriented digital services. The project’s ideation activities explored applications including climate and early-warning advisory services, smart aquaculture, soil and water monitoring, disease detection, and market intelligence ( KijaniSpace D2.2, 2026a).

Beyond technology development, KijaniSpace was conceived as a multi-actor innovation project. The Grant Agreement and project work packages incorporated stakeholder engagement as an operational component of project implementation. The planned activities included communication and dissemination, stakeholder identification and mapping, needs assessments, interviews, webinars, multi-actor workshops, ideation and co-design activities, and discussions concerning governance, partnerships and ecosystem development ( KijaniSpace Grant Agreement, 2024; KijaniSpace D2.1, 2025; KijaniSpace D2.2, 2026a; KijaniSpace D6.2, 2026b).

These activities were implemented through different work packages and at different stages of the project according to their operational objectives. Although they represented different forms of stakeholder interaction, they were not originally designed as elements of a unified conceptual framework. Instead, they supported practical purposes related to communication, contextual understanding, requirements gathering, solution development and preparation for future collaboration and use of project results.

As implementation progressed, documentary evidence accumulated through project deliverables, stakeholder mapping datasets, workshop reports, consultation records, communication activities and project reporting. These materials provide the empirical basis for the retrospective analysis presented in this paper ( KijaniSpace D2.1, 2025; KijaniSpace D2.2, 2026a; KijaniSpace D6.2, 2026b).

Accordingly, this study does not examine the implementation of a predefined stakeholder engagement framework. Instead, it examines how stakeholder engagement activities documented during KijaniSpace implementation can be retrospectively organised and interpreted using concepts derived from established stakeholder participation literature.

## 4. Analytical framework

### 4.1 Development of the analytical framework

The stakeholder engagement activities undertaken in KijaniSpace were planned as operational components of project implementation rather than as elements of a predefined conceptual model. The project included communication and dissemination, stakeholder mapping, interviews, needs assessments, webinars, workshops, ideation activities, co-design processes and collaboration-oriented discussions. These activities were implemented through different work packages and served practical purposes related to awareness-building, contextual understanding, technology development, stakeholder participation and preparation for the future use of project results.

The relationships among these activities were not originally expressed through a single stakeholder engagement framework. The analytical framework presented in this article was therefore developed retrospectively to organise and interpret the documented activities after their implementation. It should not be understood as a methodology that guided KijaniSpace from the beginning or as a model that was prospectively tested by the project.

The framework was informed by the conceptual perspectives reviewed in Chapter 2, particularly the Horizon Europe multi-actor approach, the IAP2 Spectrum of Public Participation, Agricultural Innovation Systems, participatory innovation and living lab research. These perspectives share an emphasis on interaction among different actors, the contribution of practical and contextual knowledge, and the value of participation beyond one-way communication ( Klerkx et al., 2010; Reed, 2008; Reed et al., 2018; World Bank, 2012; IAP2 Federation, n.d.).

The IAP2 Spectrum provided a useful starting point because it distinguishes among different forms of participation, including informing, consulting, involving and collaborating. However, the framework developed for this study is not presented as a replacement for, or validation of, the IAP2 Spectrum. Instead, the four categories were adapted as an analytical structure suited to the types of activities documented within KijaniSpace. The category **Empower**, which appears in the IAP2 Spectrum, was not included because the available project documentation did not demonstrate a transfer of final decision-making authority to external stakeholders.

The resulting **Multi-Actor Stakeholder Engagement Progression Framework** contains four categories:

**Inform → Consult → Involve → Collaborate.**

The term *progression* refers to differences in the form and potential depth of stakeholder participation represented by these categories. It does not imply that all stakeholders followed a fixed sequence or moved systematically from one category to the next. The project documentation does not track individual stakeholder trajectories in sufficient detail to demonstrate such movement. The categories are therefore used to classify activities rather than participants.

The framework was developed through retrospective interpretation of documented project practice. Activities were considered in relation to their principal objective, interaction format and intended stakeholder role. Communication activities were associated mainly with informing; interviews and needs assessments with consulting; workshops and co-design processes with involving; and partnership, governance and ecosystem-oriented activities with collaborating. Some activities contained characteristics of more than one category. In such cases, they were assigned to the category that best represented their principal form of interaction.

This analytical approach makes it possible to examine a heterogeneous portfolio of stakeholder activities through a common structure. At the same time, it avoids assuming that deeper engagement necessarily produced trust, ownership, institutional embedding, exploitation readiness or long-term sustainability. These outcomes were not directly measured and are therefore treated as possible implications rather than demonstrated effects.

### 4.2 Structure and interpretation of the framework

The four categories represent distinct but potentially overlapping forms of stakeholder engagement.

***Inform***

The **Inform** category includes activities whose primary purpose was to provide information, increase awareness and make project objectives, activities and results visible to relevant audiences. Typical examples include the project website, social media, newsletters, videos, webinars with a predominantly presentational format and participation in dissemination events.

In these activities, stakeholders mainly acted as recipients of information. Informing created entry points through which broader audiences could learn about the project and its technologies. However, such activities generally provided limited opportunities for stakeholders to influence project decisions or contribute contextual knowledge.

***Consult***

The **Consult** category includes activities designed to obtain stakeholder views, experiences, needs and feedback. Typical examples include interviews, surveys, stakeholder dialogues, needs assessments and consultation-oriented webinars.

Stakeholders acted primarily as knowledge contributors. Their input could help the project identify local challenges, institutional constraints, user priorities and conditions affecting the relevance or usability of proposed solutions. Consultation therefore provided a basis for contextual understanding and possible adaptation. It did not necessarily imply shared decision-making, and the extent to which stakeholder feedback influenced later project choices must be assessed from the available documentation rather than assumed.

***Involve***

The **Involve** category includes activities in which stakeholders participated more actively in defining problems, discussing priorities, generating ideas or shaping solution concepts. Examples include multi-actor workshops, ideation sessions, prioritisation exercises, roadmap development and co-design activities.

In this category, stakeholders acted as active contributors or co-creators rather than only as information recipients or consultees. Their practical, institutional and local knowledge could be brought into discussions about technology design, implementation pathways and use-case development. Involvement created opportunities for contextual learning and participatory innovation, although the available evidence does not establish that all participants acquired ownership of the resulting solutions.

***Collaborate***

The **Collaborate** category includes activities oriented towards sustained interaction, partnership development, governance dialogue, ecosystem building and continuity planning. Examples include multi-stakeholder platforms, institutional partnerships, replication discussions, policy interaction and collaboration concerning the possible exploitation or continuation of project results.

Stakeholders in this category acted as prospective or active collaborative partners. These activities extended beyond individual consultations or workshops by addressing relationships, responsibilities and possible forms of longer-term cooperation. They may have created opportunities for institutional coordination and sustainability-oriented planning. However, collaboration-oriented activity should not be interpreted as evidence that long-term partnerships, shared ownership or project continuity were already secured.

**Figure 1.**
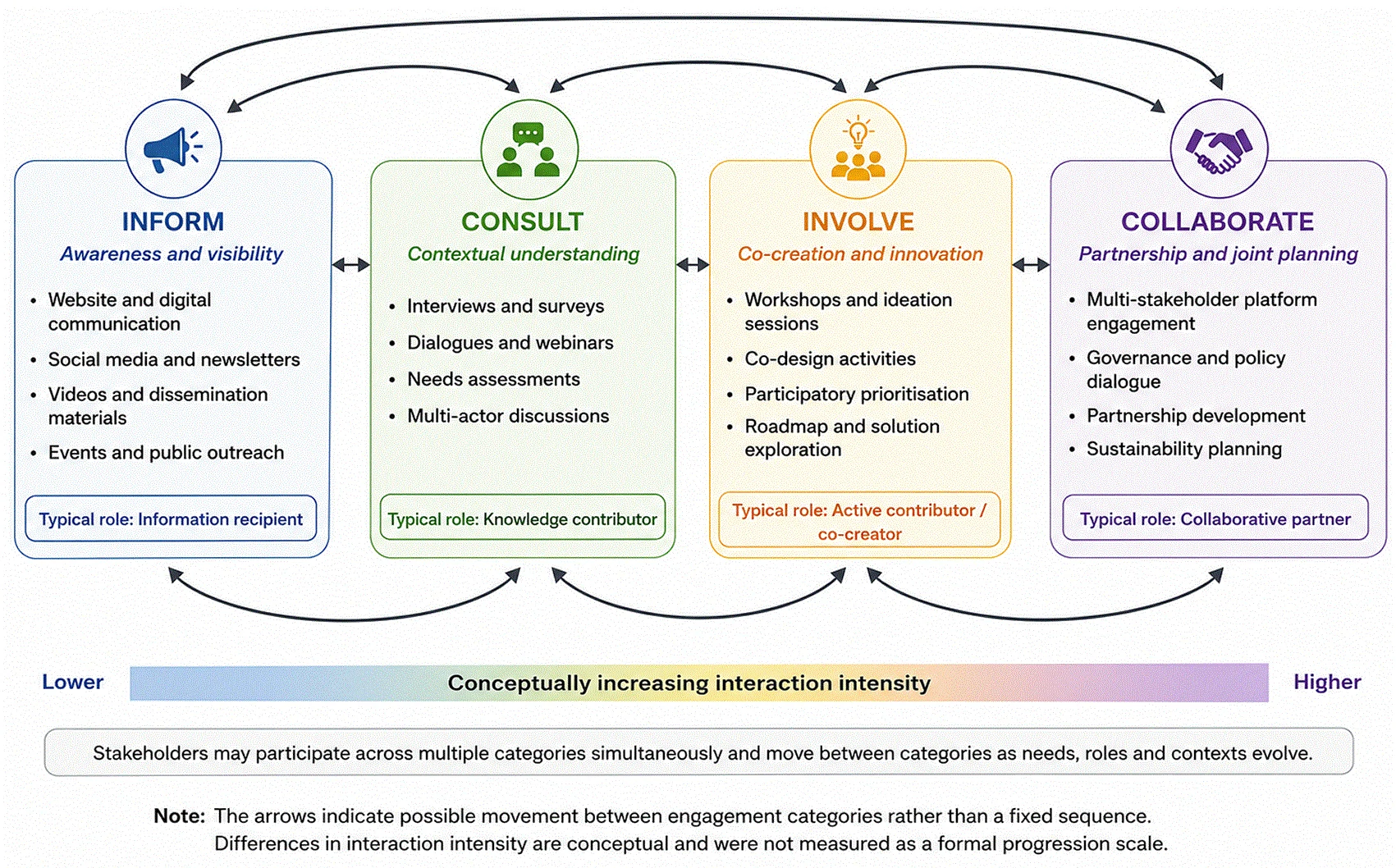
Multi-Actor Stakeholder Engagement Progression Framework used for the retrospective classification and interpretation of stakeholder engagement activities in the KijaniSpace project. The arrows indicate possible relationships between engagement categories and should not be interpreted as mandatory or sequential progression pathways.

Figure 1 illustrates the analytical framework developed through the retrospective interpretation of stakeholder engagement activities implemented within KijaniSpace. The framework organises documented activities into four conceptually distinct categories—Inform, Consult, Involve and Collaborate—according to their primary objectives and the nature of stakeholder interaction. The figure provides a visual summary of the analytical structure used throughout the remainder of this paper.

The framework should be interpreted as an analytical representation of different forms of stakeholder engagement rather than as a prescriptive implementation model. The arrows indicate that engagement activities may be related and that stakeholders may participate across multiple categories depending on project needs, stakeholder roles and contextual circumstances. They do not imply that all stakeholders move sequentially through the categories or that higher levels of interaction necessarily produce better outcomes. The horizontal axis represents conceptually increasing opportunities for interaction and contribution rather than a measured engagement scale.

Table 1 summarises the analytical framework developed through the retrospective interpretation of the KijaniSpace stakeholder engagement activities. The four categories represent analytically distinct forms of stakeholder engagement, characterised by their primary objectives, typical activities, stakeholder roles and their contribution to understanding how engagement was implemented within the project. The categories are intended as an interpretive classification rather than a prescriptive sequence of implementation.

**Table 1.** Analytical framework used to classify stakeholder engagement activities.

Category	Main objective	Typical activities	Principal stakeholder role	Analytical contribution
**Inform**	Awareness and visibility	Website, social media, newsletters, videos, dissemination events	Information receiver	Visibility, access to project information and initial awareness
**Consult**	Contextual understanding and needs identification	Interviews, surveys, dialogues, webinars, needs assessments	Knowledge contributor	Stakeholder insights, barriers, priorities
**Involve**	Co-creation and participatory innovation	Workshops, ideathons, co-design, prioritisation, roadmap development	Active contributor or co-creator	Shared concepts, pilot ideas, solution pathways
**Collaborate**	Sustainability and ecosystem continuity	Multi-stakeholder platforms, partnerships, governance interaction, replication planning	Collaborative partner	Partnership development, governance dialogue and continuity planning

The categories presented in Table 1 should not be interpreted as mutually exclusive or as mandatory stages through which all stakeholders progress. Individual engagement activities may exhibit characteristics of more than one category, and stakeholders may participate in different categories according to their roles and the objectives of specific project activities. The framework therefore provides an analytical structure for interpreting documented stakeholder engagement rather than a procedural model for implementing stakeholder participation.

## 5. Materials and methods

### 5.1 Study design

This study employs a retrospective descriptive case study design to examine stakeholder engagement activities implemented within the Horizon Europe KijaniSpace project. The objective is not to evaluate the effectiveness of stakeholder engagement or to validate a new stakeholder engagement methodology. Instead, the study retrospectively analyses documented stakeholder engagement activities and organises them using the Multi-Actor Stakeholder Engagement Progression Framework presented in Chapter 4.

Case studies are particularly appropriate for investigating complex social and organisational processes within their real-world context, especially when the boundaries between the phenomenon and its context are closely intertwined. In KijaniSpace, stakeholder engagement formed an integral part of project implementation through communication, consultation, co-creation and collaboration activities conducted across multiple work packages. These activities were designed to support project implementation and innovation uptake rather than to test a predefined conceptual framework.

The analytical framework presented in this paper was developed after the implementation of these activities through retrospective interpretation of project documentation using concepts derived from established stakeholder participation literature. Consequently, the framework serves as an analytical lens for organising and interpreting stakeholder engagement practices rather than as a prescriptive model against which project implementation is evaluated.

The study therefore adopts an interpretive rather than evaluative perspective. It seeks to answer the following research question:

**How can stakeholder engagement activities implemented within a Horizon Europe multi-actor project be retrospectively organised and interpreted using an analytical stakeholder engagement framework, and what insights does this provide into communication, consultation, involvement and collaboration during project implementation?**

The findings presented in the following sections describe how documented stakeholder engagement activities were represented within the project and how they can be interpreted using the proposed analytical framework. The study does not attempt to establish causal relationships between stakeholder engagement and outcomes such as trust, stakeholder ownership, technology adoption or long-term sustainability, as these outcomes were not systematically measured within the available project documentation.

### 5.2 Data sources

The analysis is based on documentary evidence produced during the implementation of the Horizon Europe KijaniSpace project. Documentary analysis was selected because the objective of the study is to retrospectively examine stakeholder engagement activities as they were planned, implemented and reported throughout the project. The available project documentation provides a comprehensive record of stakeholder engagement processes, their objectives, implementation and reported outcomes, making it an appropriate empirical basis for retrospective analysis.

The primary sources included the KijaniSpace Grant Agreement and Description of Action, project deliverables, periodic technical reports, communication and dissemination materials, stakeholder mapping documentation, workshop reports, needs assessments, interview summaries, co-design and ideation reports, and other project documents describing stakeholder engagement activities. Publicly available information from the project website and communication channels was also consulted where it complemented the official project documentation.

These documents collectively describe a broad range of stakeholder engagement activities, including communication and dissemination, stakeholder identification and mapping, interviews, consultations, workshops, webinars, ideation sessions, co-design activities, partnership discussions and ecosystem-building initiatives. The documentation therefore captures the diversity of stakeholder interactions implemented throughout the project and provides sufficient detail to classify activities according to the analytical framework developed in Chapter 4.

The documentary sources were not originally produced for research purposes. Instead, they were generated as part of routine project planning, implementation, reporting and communication. Consequently, the analysis presented in this paper should be understood as a retrospective interpretation of existing project documentation rather than an evaluation based on data collected specifically to test the proposed analytical framework.

### 5.3 Analytical procedure

The retrospective analysis focused on **documented stakeholder engagement activities** as the unit of analysis. Rather than analysing individual stakeholders or stakeholder groups, the study examined the engagement activities described in the project documentation and classified them according to the analytical framework presented in Chapter 4.

The documentary corpus comprised the detailed KijaniSpace multi-actor stakeholder mapping dataset and its aggregated summary tables, together with D2.1, D2.2 and D6.2. The stakeholder mapping datasets recorded stakeholder characteristics, organisational affiliation, geographic location, stakeholder group and participation across the multi-actor engagement activities implemented during 2025. The summary tables provided aggregated distributions by country, continent and stakeholder category. D2.1 documented the needs assessment, interviews, stakeholder mapping and proposed Multi-Stakeholder Platform; D2.2 documented the ideation and co-design workshops; and D6.2 consolidated communication, dissemination and stakeholder-engagement activities during the first reporting period ( KijaniSpace D2.1, 2025; KijaniSpace D2.2; 2026a, KijaniSpace D6.2, 2026b).

The author extracted each discrete stakeholder engagement activity into a coding table recording its primary objective, interaction format, intended stakeholder role, location and documentary source. Each activity was then classified according to the criteria presented in Table 1. Where an activity displayed characteristics of more than one category, the original documentary description was reviewed and the activity was assigned according to its dominant purpose. The resulting classification distinguished among Inform, Consult, Involve and Collaborate activities as defined below.

Each extracted activity was then reviewed to identify its primary objective, interaction format, intended stakeholder role and contribution to the project’s stakeholder engagement process. Based on these characteristics, activities were assigned to one of the four analytical categories of the Multi-Actor Stakeholder Engagement Progression Framework:

• **Inform** – activities primarily intended to communicate information, raise awareness and disseminate project objectives, progress or results;
• **Consult** – activities designed to obtain stakeholder knowledge, opinions, needs or feedback relevant to project implementation;
• **Involve** – activities in which stakeholders actively participated in discussions, ideation, co-design or the development of solution concepts; and
• **Collaborate** – activities focused on partnership development, governance, ecosystem building or the exploration of longer-term cooperation beyond individual project events.

The framework was applied as an **analytical classification tool** rather than as an evaluation instrument. Its purpose was to organise and interpret the diversity of stakeholder engagement activities implemented within KijaniSpace using a consistent conceptual structure. The analysis therefore describes how different forms of stakeholder engagement were represented within the project but does not assess their relative effectiveness or assume a sequential progression of stakeholders through the four categories.

The classified activities were subsequently synthesised to identify patterns of stakeholder engagement across the project and to examine how communication, consultation, involvement and collaboration contributed to the implementation of the KijaniSpace multi-actor approach. This synthesis forms the basis of the results presented in the following chapter.

### 5.4 Scope and limitations of the analysis

The analytical framework presented in this study provides a structured approach for organising and interpreting stakeholder engagement activities documented during the implementation of the KijaniSpace project. It is intended as a descriptive and interpretive tool rather than a method for evaluating stakeholder engagement performance or measuring the effectiveness of specific engagement strategies.

The analysis is based exclusively on project documentation and publicly available project outputs. Consequently, the findings reflect the information recorded during project implementation and are limited by the scope and level of detail of these documentary sources. While the documentation provides comprehensive evidence of the stakeholder engagement activities undertaken, it does not systematically capture individual stakeholder experiences, perceptions or behavioural changes over time.

Similarly, the available documentation does not provide sufficient evidence to establish causal relationships between stakeholder engagement activities and broader outcomes such as stakeholder trust, ownership, institutional embedding, technology adoption or the long-term sustainability of project results. Although these outcomes are frequently associated with effective stakeholder engagement in the literature, they were not directly measured within KijaniSpace and therefore cannot be empirically verified in this study.

The analysis also does not assume that stakeholders progressed sequentially through the four engagement categories. The categories— **Inform**, **Consult**, **Involve** and **Collaborate**—represent analytically distinct forms of stakeholder engagement rather than mandatory stages of participation. Individual activities may incorporate characteristics of more than one category, and different stakeholder groups may participate in different forms of engagement according to their roles, interests and responsibilities within the project.

Finally, this study focuses on a single Horizon Europe project. Although the analytical framework may offer a useful approach for interpreting stakeholder engagement in other multi-actor research and innovation projects, its broader applicability has not been formally evaluated. Future research could apply the framework to additional projects, compare stakeholder engagement practices across different implementation contexts, and assess its usefulness as a tool for comparative analysis.

## 6. Results

This chapter presents the results of the retrospective analysis of stakeholder engagement activities implemented within the KijaniSpace project. Using the Multi-Actor Stakeholder Engagement Progression Framework described in Chapter 4 and the analytical procedure outlined in Chapter 5, documented activities were classified according to their primary objective, form of interaction and intended stakeholder role.

The analysis identified activities corresponding to all four analytical categories: **Inform, Consult, Involve** and **Collaborate**. Together, these categories capture a range of engagement practices, from communication and dissemination to consultation, participatory co-design and collaboration-oriented initiatives. The classification provides a structured basis for examining how different forms of stakeholder engagement were represented during project implementation.

Table 2 presents selected examples of documented KijaniSpace stakeholder engagement activities classified according to the analytical framework. The analysis drew on the broader documentary corpus, while the table illustrates how representative activities were assigned to each engagement category. The examples reflect variation in activity type, stakeholder groups, geographical scope and evidence of engagement. The individual categories are examined in greater detail in Sections 6.1– 6.4.

**Table 2.** Representative examples of KijaniSpace stakeholder engagement activities classified using the analytical framework.

Gategory	Representative activity	Geographic scope	Evidence of engagement	Main stakeholder groups	Documentary source
**Inform**	Communication and dissemination activities (website, social media, newsletters, media, third-party events)	Regional and international	31,000+ website visitors; 145,000+ page views; 5,200 newsletter recipients; 55,000 LinkedIn impressions; 6,700 Facebook views; 500+ event audience	General public, researchers, SMEs, policymakers, farmers and innovation communities	D6.2
**Consult**	Needs consultations (farmer survey and key informant interviews)	Kenya, Tanzania and Uganda	**74** interview participants	Farmers, SMEs, public authorities, researchers and other local stakeholders	D2.1
**Involve**	Orientation webinar	Online/regional	**60** webinar registrations	Researchers, SMEs, innovation actors, and public authorities	D2.1/D2.2
	Ideation and co-design workshops: one in Kenya and two in Tanzania	Kenya and Tanzania	**82** workshop registrations	Researchers, SMEs, farmer organisations, public authorities and innovation actors	D2.1/D2.2
**Collaborate**	Multi-Stakeholder Platform (MSP) discussions and ecosystem-building activities	Lake Victoria Basin region	Documented activities included Multi-Stakeholder Platform discussions, ecosystem-building meetings and partnership planning.	Public institutions, SMEs, researchers, innovation hubs, farmer organisations and regional bodies	D2.1/D2.2

The results should be interpreted as a retrospective description of documented stakeholder engagement activities rather than as an evaluation of their effectiveness. The framework is used to organise and interpret the available documentary evidence and does not imply that stakeholders progressed sequentially through the four engagement categories or that all documented activities produced comparable outcomes. Instead, the analysis shows how different forms of stakeholder engagement were represented in activities related to communication, contextual understanding, participatory innovation and collaboration during the implementation of the KijaniSpace project.

### 6.1 Inform: Communication and awareness-building

The **Inform** category comprised stakeholder engagement activities whose primary objective was to communicate project information, increase visibility and raise awareness of the KijaniSpace project and its Space–IoT solutions for climate-smart agriculture and aquaculture. These activities were designed to make project objectives, technologies, events and results accessible to a broad audience while providing opportunities for stakeholders to become familiar with the project and its objectives.

The documented Inform activities included the project website, newsletters, social media communication, videos, dissemination materials, webinars with a predominantly presentational purpose and participation in external conferences and stakeholder events. The project website functioned as the principal public communication platform, providing information on project objectives, partners, news, events and publicly available project outputs. Social media channels complemented the website by extending communication to professional networks and wider stakeholder communities, while newsletters and other dissemination materials supported regular communication with interested audiences.

Project documentation indicates that these communication activities reached substantial audiences during the reporting period. As summarised in Table 2, reported communication metrics included approximately 31,000 website visitors, more than 145,000 page views, over 5,200 newsletter recipients, around 55,000 LinkedIn impressions, approximately 6,700 Facebook views and audiences exceeding 500 participants through external dissemination events. These figures demonstrate the breadth of the project’s communication activities but should be interpreted as indicators of communication reach rather than evidence of active stakeholder participation or engagement quality.

The Inform category involved a diverse range of stakeholder groups, including researchers, SMEs, policymakers, farmers, innovation communities and the general public. Communication activities created opportunities for stakeholders to access project information and become aware of ongoing developments irrespective of their geographical location or level of prior involvement in the project. As such, they contributed to the visibility of the project and provided accessible entry points for potential participation in subsequent stakeholder engagement activities.

Within the analytical framework, these activities are interpreted as representing the broadest form of stakeholder engagement. Their primary function was to disseminate information and support awareness-building rather than to obtain stakeholder knowledge or facilitate collaborative decision-making. While communication activities may encourage subsequent participation, the available documentation does not demonstrate whether stakeholders who accessed project information subsequently participated in consultation, co-design or collaboration-oriented activities. Consequently, the Inform category is interpreted as documenting communication reach and accessibility rather than progression to deeper forms of stakeholder engagement.

### 6.2 Consult: Understanding stakeholder needs and contextual conditions

The **Consult** category comprised stakeholder engagement activities designed to obtain knowledge, experiences and feedback from stakeholders to inform project implementation. Unlike the **Inform** category, which primarily focused on communication and awareness-building, consultation created opportunities for stakeholders to contribute contextual information on agricultural practices, operational challenges, institutional conditions and user requirements. The objective was to improve understanding of the regional context in which the project’s Space–IoT solutions would be developed and demonstrated.

The principal consultation activities documented within KijaniSpace included stakeholder mapping, needs assessments, structured interviews, surveys and consultation-oriented meetings conducted across Kenya, Tanzania and Uganda. These activities involved farmers, aquaculture practitioners, SMEs, researchers, public authorities, innovation intermediaries and other actors representing the regional innovation ecosystem. Together, they provided information on stakeholder priorities, existing practices, barriers to digital technology adoption and opportunities for climate-smart agriculture and aquaculture.

Stakeholder mapping formed an important component of the consultation process by identifying organisations and individuals relevant to the project’s objectives. By the project’s mid-term reporting stage, more than 200 stakeholders had been identified, representing technical users, farmer communities, policy actors, research organisations, SMEs and other innovation ecosystem participants. This mapping provided an overview of the stakeholder landscape and supported the planning of subsequent engagement activities. However, the number of mapped stakeholders should be interpreted as an indication of the breadth of the stakeholder environment rather than as evidence of active or sustained participation.

Needs assessments and interviews generated contextual information that helped characterise the operational environment for digital agriculture and aquaculture applications. The documented consultations explored issues such as farming and aquaculture practices, climate-related challenges, access to digital services, information needs and stakeholder expectations regarding Earth Observation and IoT-based solutions. These activities enabled the project consortium to gather practical knowledge from stakeholders with different institutional roles and operational experience.

Within the analytical framework, these activities are interpreted as representing consultation because their primary purpose was to obtain stakeholder knowledge rather than to develop solutions collaboratively. Stakeholders participated as **knowledge contributors**, providing information that could support contextual understanding and inform subsequent project activities. The available documentation indicates that consultation generated valuable insights into stakeholder needs, regional priorities and implementation challenges. However, it does not systematically document how individual consultation outcomes influenced specific project decisions or whether all stakeholder recommendations were subsequently incorporated into project activities.

The Consult category therefore illustrates how KijaniSpace moved beyond one-way communication by creating structured opportunities for stakeholders to contribute their knowledge and experience. Rather than representing an endpoint in the engagement process, consultation established a foundation for the more participatory activities examined in the following section, where stakeholders became active contributors to ideation, co-design and solution development through workshops and collaborative innovation activities.

### 6.3 Involve: Co-design and participatory innovation

The **Involve** category comprised stakeholder engagement activities in which participants moved beyond providing information or feedback to actively contributing to problem definition, idea generation and the development of potential solution concepts. Within KijaniSpace, these activities created opportunities for researchers, practitioners, public authorities, SMEs and other stakeholders to work together in participatory settings, where local knowledge and technical expertise could be combined to explore approaches for climate-smart agriculture and aquaculture.

The principal activities classified within this category included multi-actor workshops, ideation sessions, co-design exercises and participatory prioritisation processes conducted in Kenya and Tanzania. According to the project documentation, one workshop was organised in Kenya and two in Tanzania, with a total of 82 registered participants representing agriculture, aquaculture, research organisations, public authorities, innovation support organisations, SMEs and other regional stakeholders. These workshops were designed to encourage dialogue across stakeholder groups and to identify priorities for the development and application of Space–IoT solutions.

The workshop methodology was based on participatory and human-centred approaches. Participants discussed practical challenges affecting agriculture and aquaculture, identified priority areas for intervention and explored possible technological solutions that could address locally identified needs. The documented outputs included ideas related to climate and early-warning advisory services, smart aquaculture, soil and water monitoring, disease detection and market intelligence. These outputs illustrate how stakeholder knowledge was incorporated into the early conceptualisation and contextualisation of potential project solutions.

From the perspective of the analytical framework, these activities represent **active stakeholder involvement** because participants contributed directly to the exploration and refinement of solution concepts rather than acting solely as recipients of information or providers of feedback. The workshops created opportunities for interaction among technical experts, researchers, SMEs, public authorities and end-user representatives, enabling participants to exchange perspectives and jointly examine possible implementation pathways. In this sense, the **Involve** category reflects participatory innovation processes in which stakeholder knowledge contributes to shaping ideas and identifying contextually relevant opportunities.

The available documentation demonstrates that these participatory activities generated documented ideas, priorities and concept proposals. However, it does not systematically record whether the same stakeholders continued to participate in subsequent project activities, whether all proposed concepts were implemented, or whether participation resulted in stronger stakeholder ownership, sustained collaboration or measurable innovation outcomes. Consequently, the evidence supports the interpretation that stakeholders actively contributed to co-design and concept development, but it does not support conclusions regarding the longer-term effects of this involvement.

The **Involve** category therefore represents an important transition from consultation towards active participation in innovation processes. It demonstrates how KijaniSpace created structured opportunities for stakeholders to contribute their knowledge, experience and ideas to the development of potential Space–IoT applications. At the same time, the documentary evidence indicates that these activities should be interpreted as participatory contributions to project implementation rather than as evidence of sustained co-ownership or long-term collaborative engagement. The latter is examined in the following section through activities classified within the **Collaborate** category.

### 6.4 Collaborate: Partnership and ecosystem development

The **Collaborate** category comprised activities and emerging structures oriented towards partnership-building, governance dialogue, ecosystem coordination and possible longer-term continuity. In contrast to the **Involve** category, which focused primarily on participation in problem definition and concept development, collaboration-oriented activities addressed how stakeholder relationships, institutional roles and support structures might be maintained beyond individual workshops or consultation events. At the stage covered by the available documentation, these mechanisms had been initiated but were still under development. They should therefore be interpreted as ongoing collaboration-oriented arrangements rather than as evidence of established long-term collaboration.

The principal mechanism associated with this category was the proposed **Multi-Stakeholder Platform**. The platform was designed to support knowledge exchange, access to data and knowledge resources, collaboration among start-ups, SMEs, researchers and institutions, and the integration of traditional and indigenous knowledge with modern innovations. Project documentation described it as a flexible and context-sensitive mechanism intended to support broad stakeholder representation, farmer participation, feedback and peer learning.

The collaboration-oriented structure also included planned **Technical Working Groups** covering policy, research and innovation, and application. These groups were intended to create opportunities for interaction among policymakers, researchers, farmers, NGOs and other ecosystem actors. Their anticipated functions included supporting transparent discussion, capacity-building and coordination around future implementation. However, their development remained ongoing during the period examined, and the available documentation does not provide evidence that they had yet become fully established governance structures.

Further collaboration-oriented activities were linked to institutional integration, regional and government partnerships, monitoring and evaluation, knowledge repositories, replication planning and longer-term ecosystem support. These elements indicate that KijaniSpace was seeking to connect stakeholder engagement with governance arrangements, future implementation pathways and sustainability planning. Public authorities, innovation hubs, researchers and SMEs also participated in discussions concerning partnerships, ecosystem continuity, pilot implementation and possible replication opportunities.

Within the analytical framework, these activities are classified as **Collaborate** because their main purpose extended beyond communication, consultation and co-design towards the development of relationships and structures for future cooperation. Stakeholders were positioned not only as recipients of information or contributors of knowledge, but as prospective institutional and ecosystem partners involved in discussions about governance, coordination and continuity.

Nevertheless, the documentary evidence supports only a cautious interpretation of this category. The available materials demonstrate that collaboration-oriented discussions, structures and partnership activities were planned or ongoing, but they do not establish their effectiveness or long-term continuity. In particular, the evidence does not demonstrate institutional embedding, sustained partnership performance, collaborative ownership, exploitation readiness or ecosystem sustainability. These outcomes would require longitudinal monitoring and evidence collected beyond the period analysed in this study.

The **Collaborate** category therefore captures the project’s efforts to create opportunities for continued institutional interaction and ecosystem development. It shows that stakeholder engagement within KijaniSpace extended beyond individual communication, consultation and co-design activities to include planning for partnership, governance and future coordination. At the same time, it remains important to distinguish the presence of collaboration-oriented mechanisms from demonstrated long-term collaborative outcomes.

## 7. Discussion

### 7.1 Interpretation of stakeholder engagement within KijaniSpace

The retrospective analysis demonstrates that stakeholder engagement within KijaniSpace encompassed a diverse set of communication, consultation, participatory and collaboration-oriented activities that can be meaningfully organised using the Multi-Actor Stakeholder Engagement Progression Framework. Rather than representing isolated engagement events, the documented activities illustrate how different forms of stakeholder interaction supported complementary objectives throughout project implementation.

The analysis indicates that communication and dissemination activities created broad awareness of the project and its objectives, while consultation activities generated contextual knowledge concerning stakeholder needs, local conditions and implementation challenges. Participatory workshops and co-design activities provided opportunities for stakeholders to contribute ideas, discuss priorities and explore potential applications of Space–IoT technologies. Collaboration-oriented initiatives extended stakeholder engagement towards partnership development, ecosystem building and governance discussions.

These findings suggest that stakeholder engagement within a Horizon Europe multi-actor project is better understood as a portfolio of complementary engagement approaches than as a single participation process. Different activities served different implementation needs, involved different stakeholder groups and required different forms of interaction. Consequently, the four categories should be interpreted as complementary analytical perspectives rather than successive implementation stages.

The retrospective analysis also indicates that stakeholder engagement was iterative and context-dependent. Activities frequently overlapped, and stakeholders could participate in different forms of engagement according to their expertise, institutional responsibilities and opportunities for participation. The available documentation does not support the interpretation that stakeholders progressed sequentially through the four engagement categories. Instead, the concept of progression reflects increasing opportunities for stakeholder interaction and contribution rather than measured participant trajectories.

### 7.2 Relationship to existing stakeholder engagement literature

The findings are consistent with previous research emphasising that innovation in agriculture emerges through interaction among multiple actors rather than through linear technology transfer ( Klerkx et al., 2010; World Bank, 2012). The consultation and co-design activities documented within KijaniSpace illustrate how contextual knowledge from farmers, researchers, SMEs, public authorities and other stakeholders contributed to discussions concerning the design and implementation of digital agriculture solutions.

Similarly, the results align with participatory innovation and living lab research, which emphasises iterative learning, stakeholder participation and the integration of practical knowledge into innovation processes ( Reed et al., 2018; Toffolini et al., 2023; Gardezi et al., 2024). However, the KijaniSpace case also demonstrates that participation within large international research projects is distributed across multiple activity types rather than confined to formal co-design workshops. Communication, consultation, involvement and collaboration each fulfilled different functions within the broader implementation process.

The analytical framework also draws inspiration from the IAP2 Spectrum of Public Participation, particularly the distinction between informing, consulting, involving and collaborating stakeholders. Unlike the IAP2 Spectrum, however, the framework presented in this study was not designed as a normative participation model. Instead, it was developed retrospectively as an interpretive structure for classifying and analysing documented stakeholder engagement activities within a Horizon Europe multi-actor project. In this respect, the framework complements rather than replaces existing stakeholder participation models by demonstrating one possible approach to organising project documentation for analytical purposes.

### 7.3 Implications for Horizon Europe multi-actor projects

The study has practical implications for the planning, documentation and evaluation of stakeholder engagement within Horizon Europe projects. Many multi-actor projects implement a broad range of engagement activities but document them primarily as individual project tasks or deliverables. Retrospective analysis using a common analytical framework can help reveal relationships among these activities and provide a more coherent understanding of how different forms of stakeholder engagement contribute to project implementation.

The framework may also support project monitoring and comparative analysis by providing a consistent vocabulary for describing stakeholder engagement activities. Rather than focusing exclusively on communication outputs or participation numbers, projects may benefit from documenting the objectives, interaction formats and intended stakeholder roles associated with different engagement activities. Such documentation could facilitate cross-project learning and contribute to a more systematic understanding of stakeholder engagement practices within Horizon Europe.

At the same time, the framework should not be interpreted as a prescriptive implementation model. Different projects operate in different institutional, technological and socio-economic contexts, and stakeholder engagement strategies should remain adaptable to project objectives, stakeholder capacities and regional circumstances. The framework therefore offers an analytical structure that may support reflection and comparison rather than a standardised methodology for stakeholder engagement.

### 7.4 Limitations and future research

Several limitations should be considered when interpreting the findings. First, the study is based on documentary evidence from a single Horizon Europe project. Although the documentation provides a comprehensive record of stakeholder engagement activities, it was produced primarily for project management and reporting rather than for research purposes.

Second, the retrospective analysis classifies documented activities rather than evaluating stakeholder experiences or engagement outcomes. The available evidence does not allow conclusions to be drawn regarding changes in stakeholder attitudes, trust, ownership, technology adoption or the long-term sustainability of project results. Nor does it permit the reconstruction of individual stakeholder participation across successive engagement activities.

Third, the analytical framework has been applied only to the KijaniSpace project. Its broader usefulness for analysing stakeholder engagement in other research and innovation projects remains to be examined.

Future research could apply the framework to additional Horizon Europe and other multi-actor projects, compare stakeholder engagement patterns across different thematic domains and implementation contexts, and combine documentary analysis with interviews, surveys or longitudinal participant tracking. Such studies would help assess the framework’s wider applicability and provide stronger evidence concerning the relationships between stakeholder engagement processes and project outcomes.

## 8. Conclusions

This study retrospectively examined stakeholder engagement activities implemented within the Horizon Europe KijaniSpace project and organised them using the **Multi-Actor Stakeholder Engagement Progression Framework** developed through the retrospective interpretation of project documentation. Rather than proposing a new stakeholder engagement methodology, the study demonstrates how documented stakeholder engagement activities can be systematically classified and interpreted using concepts derived from established stakeholder participation literature.

The analysis showed that stakeholder engagement within KijaniSpace extended beyond communication and dissemination to include consultation, participatory involvement and collaboration-oriented activities. These activities served different but complementary purposes during project implementation, including raising awareness, gathering contextual knowledge, supporting co-design and exploring partnership and ecosystem development. The retrospective application of the analytical framework provided a coherent structure for describing these different forms of engagement while recognising that stakeholder participation was context-dependent, overlapping and not necessarily sequential.

The principal contribution of this study is the development of an analytical approach for interpreting stakeholder engagement activities within a Horizon Europe multi-actor project. By organising documented activities into the categories **Inform**, **Consult**, **Involve** and **Collaborate**, the framework provides a consistent vocabulary for describing and comparing stakeholder engagement practices. As an interpretive framework, it may support project reflection, documentation and comparative analysis across research and innovation projects, while complementing established stakeholder participation concepts such as the Horizon Europe multi-actor approach, the IAP2 Spectrum of Public Participation and Agricultural Innovation Systems.

The study also highlights the importance of documenting stakeholder engagement in a manner that captures not only communication outputs but also the objectives, forms of interaction and stakeholder roles associated with different engagement activities. Such documentation can improve the transparency of stakeholder engagement processes and facilitate learning across projects implementing multi-actor approaches.

Several limitations should be recognised. The framework was developed through retrospective analysis of a single Horizon Europe project and was applied using documentary evidence generated during project implementation. Consequently, the study does not evaluate the effectiveness of stakeholder engagement or establish causal relationships between engagement activities and outcomes such as stakeholder trust, ownership, technology adoption or long-term sustainability. These outcomes require dedicated empirical investigation using participant-based and longitudinal research approaches.

Future research should apply the framework to additional multi-actor projects in different thematic and geographical contexts to examine its broader applicability and support comparative analyses of stakeholder engagement practices. Combining documentary analysis with interviews, surveys and longitudinal stakeholder monitoring would also provide a stronger basis for evaluating how different forms of stakeholder engagement contribute to innovation processes and project outcomes.

### Ethics and consent

This study is based on the retrospective analysis of project documentation generated during the implementation of the Horizon Europe KijaniSpace project. The analysis used project reports, public deliverables, communication materials and other documentary sources describing stakeholder engagement activities. No new data were collected directly from human participants for the purposes of this study.

Where stakeholder engagement activities involved interviews, workshops or surveys conducted as part of the KijaniSpace project, these activities were implemented within the project’s established ethical procedures and applicable institutional and Horizon Europe requirements. The present study analyses the documented outputs of these activities and does not report personally identifiable information.

### Use of generative AI tools

Generative artificial intelligence (AI) tools were used to support language editing, improve clarity and assist in the organisation of the manuscript. The authors reviewed, revised and verified all generated text and remain fully responsible for the content, interpretation and conclusions presented in this article. Generative AI tools were also used to support the preparation of illustrative figures. All figures were subsequently reviewed and finalised by the authors.

## Data Availability

The analysis is based on documentation produced during the Horizon Europe KijaniSpace project. Publicly available sources include the KijaniSpace project website, public project deliverables and communication and dissemination materials. The identifiable stakeholder mapping dataset is not publicly available because it contains personal data and potentially identifiable organisational information. Aggregated stakeholder distributions and the project-level evidence used in the analysis are reported in the article and cited public deliverables. Access to any non-public material is subject to applicable data-protection requirements, participant consent and consortium approval. Publicly available project materials can be accessed through the KijaniSpace project website and the project’s Zenodo community.
