# Supplementary material for: Retrospective Analysis of Stakeholder Engagement Activities in a Horizon Europe Multi-Actor Project: Evidence from KijaniSpace
Source: Open Res Eur. 2026 Aug 21;6:193. Originally published 2026 Jun 4. [Version 2] doi: 10.12688/openreseurope.23950.2 (PMC13616056; doi:10.12688/openreseurope.23950.2)
Supplement: Peer review history

# Reviewer report: Reviewer response for version 2

**DOI:** 10.21956/openreseurope.26741.r79624

**Published:** 2026-09-26

**Authors:** Matthew Ayamga

**Affiliations:** 1 Wageningen University, Wageningen, The Netherlands

I found the paper interesting because of my experience in EU projects focused on stakeholder engagement and collaboration in co-identifying and co-creating/designing solutions.

 Also, most of my comments were already addressed in the earlier round of revisions.

 However, my main comment concerns how the gap is framed. EU projects I know usually set out in the grant agreement how stakeholder engagement activities will be carried out, so why should this require an explicit conceptual stakeholder framework when existing research already offers a range of stakeholder engagement approaches?

 I would expect the gap framing to point to whether the project's stakeholder engagement activities follow any known approaches, or to a new way to reconceptualize them. The current framing is close to this and can be easily addressed.

Is the case presented with sufficient detail to be useful for teaching or other practitioners?

Yes

Is the work clearly and accurately presented and does it cite the current literature?

Yes

If applicable, is the statistical analysis and its interpretation appropriate?

Not applicable

Are all the source data underlying the results available to ensure full reproducibility?

Yes

Are the conclusions drawn adequately supported by the results?

Yes

Is the background of the case’s history and progression described in sufficient detail?

Yes

Reviewer Expertise:

Innovation management, interdisciplinary collaboration, agricultural innovation ecosystems, entrepreneurship and innovation, business management

I confirm that I have read this submission and believe that I have an appropriate level of expertise to confirm that it is of an acceptable scientific standard.

# Reviewer report: Reviewer response for version 2

**DOI:** 10.21956/openreseurope.26741.r80406

**Published:** 2026-09-25

**Authors:** Yiorgos Gadanakis

**Affiliations:** 1 Cyprus University of Technology, Limassol, Cyprus

It was really interesting reading this manuscript and its approach. Based on previous comments and reviews, the author has successfully repositioned it from an over-ambitious "new methodology" paper to a retrospective case study, which is much better aligned with the available evidence. The paper is coherent, transparent about its limitations, and generally well written although, there are sections that are repeated across the manuscript - especially statements about the objective of the paper and the treatment of methodology. I believe that this repetition can be limited. 

 Examples:

 The manuscript repeatedly states:

- "Retrospective descriptive case study"
- "Activities rather than stakeholders"
- "Not a validated methodology"
- "No causal relationships are claimed"

These phrases appear many times across Introduction, Methods, Results, Discussion and Conclusions

 Overall, the main strengths of the paper can be summarized around the structure of a clear research question, an appropriate case study design, and a transparent description of data sources. Moreover, it demonstrates a good integration with the IAP2, AIS and living lab literature and is also demonstrating practical relevance for Horizon Europe multi actor approach projects. 

 However, a major concern remains as it is also described from another reviewer and that is of the use of the term "progression" since this creates a conceptual inconsistency. Throughout the manuscript, it is repeated that activities are classified and not people, no stakeholder trajectories were tracked and that no sequential movement was demonstrated. Yet the framework is still mentioned as a Multi-Actor Stakeholder Engagement Progression Framework. Therefore, the author might need to consider revisiting the title of the framework and avoid the use of the word progression. 

 The Results section could briefly summarize the main patterns emerging from the analysis, rather than focusing primarily on describing activities within each category.

 In the discussion section, I would encourage for a stronger critical reflection of the findings, for example, in the discussion, the author could focus the effort in answering the questions below:

- Why was Collaborate the least evidenced category?
- What barriers prevented deeper engagement?
- What are the risks of relying on project-generated documentation?
- How do Horizon Europe reporting requirements shape stakeholder engagement evidence?

Based on the response of the authors to comments from reviewers and also the extensive revision of the manuscript from its original format I could suggest that the paper is now indexable, but it would be stronger if it shifted from describing activities to analyzing patterns emerging from those activities and resolved the remaining conceptual inconsistency around "progression."

Is the case presented with sufficient detail to be useful for teaching or other practitioners?

Yes

Is the work clearly and accurately presented and does it cite the current literature?

Yes

If applicable, is the statistical analysis and its interpretation appropriate?

Not applicable

Are all the source data underlying the results available to ensure full reproducibility?

No source data required

Are the conclusions drawn adequately supported by the results?

Yes

Is the background of the case’s history and progression described in sufficient detail?

Yes

Reviewer Expertise:

I have worked in a number of Horizon funded projects as the leader of co-production activities, stakeholder engangement and multi actor approach designing frameworks.

I confirm that I have read this submission and believe that I have an appropriate level of expertise to confirm that it is of an acceptable scientific standard, however I have significant reservations, as outlined above.

# Reviewer report: Reviewer response for version 2

**DOI:** 10.21956/openreseurope.26741.r79562

**Published:** 2026-09-01

**Authors:** Franco Silveira

**Affiliations:** 1 Embrapa Digital Agriculture, Campinas, State of São Paulo, Brazil

Thank you for the careful and substantial revision of the manuscript, as well as for the detailed responses to the reviewers’ comments.

 The revised version is considerably clearer in its methodological positioning and presents the study much more coherently as a retrospective descriptive case study. I would also like to acknowledge the effort made to clarify the relationship with existing participation frameworks, strengthen methodological transparency, and moderate claims that were not directly supported by the available evidence.

 I believe the manuscript has improved substantially. However, there is one remaining point that I suggest considering before finalising the article.

 The designation “Multi-Actor Stakeholder Engagement Progression Framework”, together with the graphical representation “Inform → Consult → Involve → Collaborate”, may still convey the idea of a sequential progression or movement across different levels of engagement. At the same time, the revised version explicitly clarifies that individual stakeholder trajectories were not tracked, that sequential progression was not demonstrated, and that the categories may overlap or occur independently.

 Given this revised conceptual positioning, I suggest reconsidering whether the term “Progression” and the use of directional arrows are fully consistent with the empirical evidence and with the analytical purpose of the framework.

 A more neutral designation, such as “Multi-Actor Stakeholder Engagement Analytical Framework” or “Multi-Actor Stakeholder Engagement Classification Framework”, could more accurately reflect the retrospective, analytical, and non-sequential nature of the study. Alternatively, the authors could provide a stronger conceptual justification for retaining the term “Progression”.

 Overall, I appreciate and acknowledge the substantial improvements made in this revised version of the manuscript.

Reviewer Expertise:

Digital Agriculture; Agriculture 4.0; Innovation Ecosystems; Agricultural Innovation Systems; Technology Adoption; Agri-food Systems.

I confirm that I have read this submission and believe that I have an appropriate level of expertise to confirm that it is of an acceptable scientific standard, however I have significant reservations, as outlined above.

# Reviewer report: Reviewer response for version 1

**DOI:** 10.21956/openreseurope.25930.r76422

**Published:** 2026-06-30

**Authors:** Vida Dabkienė

**Affiliations:** 1 Lithuanian Centre for Social Sciences, Vilnius, Lithuania

The manuscript addresses a highly relevant topic and presents an interesting attempt to conceptualize stakeholder engagement within a Horizon Europe innovation project. The practical experience gained from the KijaniSpace project clearly represents a valuable foundation for developing such a framework, and the manuscript successfully demonstrates the complexity of stakeholder engagement in AU–EU innovation ecosystems.

 At the same time, as this is presented as a methodological paper, I expected a more detailed description of how the proposed method was actually developed and operationalized. The manuscript provides an extensive conceptual discussion and numerous examples of project activities; however, the methodological development itself remains insufficiently transparent. In particular, it would considerably strengthen the paper if the authors described the development process in greater detail. For example, it would be helpful to know how many experts or project partners contributed to the framework, whether the model evolved through several iterations, how the engagement stages were defined and refined, and what criteria were used to classify activities into the proposed categories. Likewise, more information about the workshops, stakeholder consultations, participant profiles, and analytical procedures would allow readers to better understand the methodological logic behind the framework.

Are sufficient details provided to allow replication of the method development and its use by others?

Partly

Is the description of the method technically sound?

Yes

Are the conclusions about the method and its performance adequately supported by the findings presented in the article?

Partly

Is the rationale for developing the new method (or application) clearly explained

Yes

If any results are presented, are all the source data underlying the results available to ensure full reproducibility?

Partly

Reviewer Expertise:

Application of the Living Lab Methodology for Agricultural Innovation

I confirm that I have read this submission and believe that I have an appropriate level of expertise to state that I do not consider it to be of an acceptable scientific standard, for reasons outlined above.

# Response

**Published:** 2026-07-10

**Authors:** Tuula-Maija Löytty

Dear Vida Dabkienė, 

 First of all, I would like to sincerely thank You for the highly knowledgeable, thoughtful and constructive review. I greatly appreciate the time and expertise devoted to reading the manuscript and providing such clear and valuable comments. I am particularly grateful for the positive assessment of the practical relevance of the work and for recognising the value of the experiences gained during the KijaniSpace Horizon Europe project.

 My original intention was to document these practical experiences and to organise them into a structured stakeholder engagement approach that could be useful for similar innovation ecosystems. After carefully considering your comments, I realised that **I positioned the manuscript incorrectly by presenting it as a methodological paper**.

 This was largely due to my own misunderstanding of the scientific expectations associated with the term *model*. My professional background is primarily in leading and implementing European innovation projects rather than in developing formal scientific methods, and I underestimated the methodological requirements implied by presenting the work as a new model. My intention was never to claim the development of a universally applicable or fully validated stakeholder engagement model. Rather, I sought to describe and synthesise a practical approach that emerged during the implementation of the KijaniSpace project. Looking back, terms such as *approach*, *framework*, or *practice pattern* would have been more appropriate than *model*. Your comments regarding the need for a much more transparent description of the development process, the iterative refinement of the framework, stakeholder participation, analytical procedures and operationalisation are entirely justified. They have helped me recognise that the manuscript, in its current form, does not meet the expectations of a methodological paper.

 After reflecting on the reviews, I believe that the manuscript would benefit from a fundamentally different positioning. Rather than attempting to revise it as a Method Article, I believe it would be more appropriate to redevelop it in the future as an exploratory case study describing stakeholder engagement practices and lessons learned from the KijaniSpace project, with the proposed framework presented as an empirical synthesis rather than as a new methodological model. I would like to thank you once again for your constructive and insightful review. Your comments have significantly improved my understanding of how practical innovation outcomes developed within European research and innovation projects should be positioned and communicated in scientific publications, and they will undoubtedly influence my future research writing.

 Tuula

# Response

**Published:** 2026-07-22

**Authors:** Tuula-Maija Löytty

We thank Reviewer 2 for the constructive assessment and for recognising the value of the practical experience generated within KijaniSpace. The reviewer’s main concern was that the article was presented as a methodological paper without sufficient transparency concerning how the method was developed and operationalised. We agree that the original framing and evidence were not adequately aligned.

**1. Status and development of the framework** **Comment:** The manuscript should provide a clearer description of how the proposed method was developed.

**Response:** The revised manuscript clarifies that KijaniSpace did not prospectively develop and implement the framework as a project methodology. Communication, mapping, consultations, workshops, co-design and collaboration-oriented activities had been planned in the Grant Agreement as operational tasks. The framework was developed afterwards through retrospective interpretation of the documented activities using concepts from IAP2, Agricultural Innovation Systems, living labs and multi-actor research. This chronology is now stated explicitly in the Introduction, KijaniSpace context, analytical framework and methods. The manuscript therefore no longer claims that the framework guided implementation or was tested as a predefined method.

**2. Expert involvement and iterative refinement** **Comment:** The manuscript should state how many experts or partners developed the model and whether it evolved through several iterations.

**Response:** Because the framework was not created through a formal expert-consensus or iterative method-development process, the revised article does not imply that such a process occurred. It identifies the author’s retrospective documentary analysis and explains the theoretical sources and classification logic used. We considered it more transparent to state the actual analytical process than to reconstruct a methodological development history that was not documented. The absence of formal multi-expert development and independent validation is reflected in the limitations and in the decision to present the framework as interpretive rather than validated.

**3. Definition and classification of engagement categories** **Comment:** The criteria used to define, refine and assign activities to the categories should be explained.

**Response:** Section 5.3 now gives the classification procedure in detail. Each discrete activity was extracted into a coding table recording its primary objective, interaction format, intended stakeholder role, location and documentary source. Activities were classified according to the definitions in Table 1. Where an activity displayed characteristics of more than one category, the original documentary description was reviewed and the activity was assigned according to its dominant purpose. The revised framework also clarifies that categories classify activities rather than participants and are not mutually exclusive or mandatory sequential stages.

**4. Documentary sources and analytical corpus** **Comment:** More detail was needed about the material used to derive the analysis.

**Response:** The revised methods identify the principal corpus as:

- the detailed KijaniSpace multi-actor stakeholder mapping dataset;
- aggregated stakeholder mapping summaries;
- D2.1, covering needs assessment, interviews, mapping and the proposed Multi-Stakeholder Platform;
- D2.2, covering ideation and co-design workshops;
- D6.2, covering communication, dissemination and stakeholder-engagement activities during the first reporting period.

The broader project documentation, Grant Agreement, periodic reports and public communication materials are described in Section 5.2 as contextual and supporting sources. **5. Workshops, consultations and participant profiles** **Comment:** Additional information was requested concerning workshops, stakeholder consultations and participant profiles. **Response:** The revised Results provide representative activities, geographical scope, documented participation or reach, stakeholder groups and source documents. Examples include interview participants, webinar registrations, workshop registrations, communication reach and collaboration-oriented activities. The manuscript distinguishes between mapped stakeholders, event registrations, actual consultation participants and public communication reach. It also states that these figures are not directly comparable and do not measure engagement quality or individual progression.

**6. Reproducibility** **Comment:** Greater methodological transparency was required to allow replication.

**Response:** The revised manuscript provides a reproducible logic for applying the analytical framework to another documentary corpus: identify discrete engagement activities, record their objectives, interaction formats and stakeholder roles, and classify them according to Table 1 using dominant purpose where categories overlap. At the same time, the manuscript now accurately limits the reproducibility claim. The source documentation was produced for project management and reporting rather than as a predesigned research dataset, and some identifiable stakeholder information cannot be released. The study is therefore reproducible at the level of analytical procedure and public documentary evidence, but not as a fully open participant-level dataset.

**7. Support for conclusions** **Comment:** The conclusions concerning the performance of the method were only partly supported.

**Response:** The conclusions have been rewritten so that they no longer make performance claims. The revised article concludes only that the four-category framework provides a coherent way to organise heterogeneous documented activities. It does not conclude that the framework improved engagement or produced trust, ownership, sustainability or exploitation readiness. The manuscript now explicitly identifies longitudinal tracking, interviews, surveys, independent stakeholder feedback and comparative application across projects as necessary for evaluating such outcomes.

# Reviewer report: Reviewer response for version 1

**DOI:** 10.21956/openreseurope.25930.r76418

**Published:** 2026-06-29

**Authors:** Franco Silveira

**Affiliations:** 1 Embrapa Digital Agriculture, Campinas, State of São Paulo, Brazil

The manuscript addresses a highly relevant topic, particularly in the context of digital agriculture, innovation ecosystems, climate-smart agriculture, and AU/EU collaboration initiatives. The article is well written, logically structured, and provides valuable practical insights from the KijaniSpace project.

 However, while the practical relevance of the work is evident, several aspects require further clarification and strengthening before the proposed framework can be considered a robust methodological contribution.

 Major Comments:

 Originality of the framework - The manuscript presents the multi-actor stakeholder engagement progression model as a novel methodological contribution. However, the proposed framework closely resembles existing stakeholder participation models, particularly the IAP2 spectrum of public participation and related participatory governance frameworks. The authors should more clearly articulate: what is genuinely novel in the proposed model, how it differs conceptually and operationally from existing participation frameworks, and why a new framework is needed instead of adapting existing approaches. At present, the distinction from prior models remains insufficiently demonstrated.

 Concepts - The manuscript frequently combines the concepts of Digital Agriculture and Climate-Smart Agriculture, often treating them as complementary or closely intertwined approaches. However, these concepts have distinct theoretical origins, objectives, and scopes. Although there is an important overlap between them, the manuscript does not clearly explain how these approaches are related within the proposed framework. In some sections, digital agriculture appears to be presented as a tool supporting climate-smart agriculture, while in others the concepts appear to be used almost interchangeably.

 I recommend that the authors provide a clearer conceptual discussion, preferably in the Introduction or Background section, addressing:

 - What is meant by Digital Agriculture;

 - What is meant by Climate-Smart Agriculture;

 - How these concepts interact within the KijaniSpace project;

 - The specific role of Space–IoT technologies in connecting these two approaches.

 A stronger conceptual foundation would improve the theoretical coherence of the manuscript and reduce potential ambiguity for readers.

 Limited empirical validation - The manuscript primarily relies on descriptive evidence derived from project activities, stakeholder interactions, workshops, consultations, and communication efforts. Although these examples illustrate framework implementation, they do not constitute strong validation of the framework itself. The manuscript would benefit from: explicit evaluation criteria, indicators measuring stakeholder progression, and evidence demonstrating improved outcomes compared to alternative engagement approaches. Currently, many conclusions remain largely inferential.

 Reproducibility and transparency - The article references project deliverables (e.g., D2.1, D2.2, D6.2) as major sources of evidence. However, sufficient methodological details are not always provided within the manuscript itself. The authors should provide additional information regarding stakeholder sampling procedures, number and profiles of participants, workshop methodologies, data collection procedures, and analytical methods used to derive conclusions. This would improve reproducibility and methodological transparency.

 Observations on clarity, precision, and conceptual consistency - Several statements throughout the manuscript are overly broad and generic, reducing the scientific rigor of the arguments presented.

 For example, the Introduction states: *"This is particularly important in innovation ecosystems that combine emerging digital technologies, environmental monitoring and local development objectives across diverse institutional and socio-economic contexts". *However, it remains unclear which institutional and socio-economic contexts are being discussed. Does this refer specifically to East Africa, the Lake Victoria Basin, low-income countries, or agricultural systems more broadly? Greater contextualization is needed.

 Similar issues can be observed in several other statements, including:

*"Stakeholder engagement is increasingly recognised as a critical component of innovation governance..."* — Recognized by whom?

*"Recent research and innovation programmes increasingly recognise..."* — Which programmes? European, African, or international?

*"The findings suggest that progressively deepening stakeholder engagement can strengthen collaborative ownership..."* — What empirical evidence supports this claim?

*"The framework may have broader applicability beyond the specific Lake Victoria Basin context"* — Which contexts specifically?

 Additionally, concepts such as *trust-building*, *ownership*, *ecosystem continuity*, *adaptive governance*, and *sustainability* are frequently used without clear operational definitions or measurable indicators. The manuscript would benefit from more precise, contextualized, and evidence-based language throughout.

 Conclusions - Several conclusions suggest that the framework strengthened trust, ownership, sustainability, adaptive governance, and exploitation readiness. While these claims are plausible, the manuscript currently provides limited empirical evidence demonstrating these outcomes. The authors should either provide stronger supporting evidence or moderate the language used in the results and conclusions sections to better reflect the descriptive nature of the study.

 Minor Comments:

 Figure 1 provides a useful visual summary of the framework but remains relatively simplistic for a methodological paper. The inclusion of additional theoretical constructs, causal mechanisms, or relationships between framework components would strengthen the contribution.

 A comparative table contrasting the proposed framework with existing stakeholder engagement frameworks would help clarify its unique contribution.

 The limitations section could more explicitly discuss potential biases associated with self-assessment within an ongoing Horizon Europe project.

 The manuscript would benefit from a dedicated discussion of the indicators used to assess stakeholder progression across engagement levels.

 - The manuscript addresses an important and timely topic and presents valuable practical experiences from a large international innovation project. However, its scientific contribution would be significantly strengthened through a clearer demonstration of originality, stronger empirical validation, greater methodological transparency, and a more cautious interpretation of the results. Therefore, I recommend publication only after substantial revisions addressing the concerns outlined above.

Are sufficient details provided to allow replication of the method development and its use by others?

Partly

Is the description of the method technically sound?

Yes

Are the conclusions about the method and its performance adequately supported by the findings presented in the article?

Partly

Is the rationale for developing the new method (or application) clearly explained

Partly

If any results are presented, are all the source data underlying the results available to ensure full reproducibility?

Partly

Reviewer Expertise:

Digital Agriculture; Agriculture 4.0; Innovation Ecosystems; Agricultural Innovation Systems; Technology Adoption; Agri-food Systems.

I confirm that I have read this submission and believe that I have an appropriate level of expertise to state that I do not consider it to be of an acceptable scientific standard, for reasons outlined above.

## References

[1] International Association for Public Participation (IAP2), 2018. IAP2 Spectrum of Public Participation. Denver, CO: IAP2. Available at: https://cdn.ymaws.com/www.iap2.org/resource/resmgr/pillars/spectrum_8.5x11_print.pdf [Accessed 24 Jun. 2026].

[2] Leminen, S., Westerlund, M., & Nyström, A. G. (2012). Living Labs as open-innovation networks. Technology Innovation Management Review, 2(9), 6–11.

[3] : Achieving inclusive digital development: A frugal strategy based on lessons from three West African smallholder agriculture value chains. *Technological Forecasting and Social Change* .2025;220: 10.1016/j.techfore.2025.124287 10.1016/j.techfore.2025.124287

[4] : Towards a theory of ecosystems. *Strategic Management Journal* .2018;39(8) : 10.1002/smj.29042255-2276 10.1002/smj.2904

[5] : Silicon Savannah and smallholder farming: How can digitalization contribute to sustainable agricultural transformation in Africa?. *Agricultural Systems* .2025;222: 10.1016/j.agsy.2024.104180 10.1016/j.agsy.2024.104180

[6] : Developing a framework for responsible innovation. *Research Policy* .2013;42(9) : 10.1016/j.respol.2013.05.0081568-1580 10.1016/j.respol.2013.05.008

# Response

**Published:** 2026-07-10

**Authors:** Tuula-Maija Löytty

Dear Franco Silveira

 First of all, I would like to sincerely thank You for the exceptionally knowledgeable, thoughtful and constructive review. I greatly appreciate the time and expertise invested in reading the manuscript and providing such detailed comments and relevant literature recommendations. After carefully reading the review, I realised that the main weakness of my manuscript is not the practical work itself, but the way I positioned its scientific contribution. **This was largely due to my own misunderstanding of the scientific implications of presenting the work as a **model**.**

 My professional background is primarily in leading and implementing European innovation projects rather than in developing formal scientific methods. Consequently, I underestimated the methodological expectations associated with the term *model* in the scientific literature.

 My original intention was not to propose a universally valid stakeholder engagement model. Rather, I wanted to document and structure a stakeholder engagement approach that proved useful during the implementation of the KijaniSpace project. In retrospect, terms such as *approach*, *framework*, or even *practice pattern* would probably describe the contribution more accurately than *model*. The review has been extremely valuable because it has helped me better understand the distinction between practical innovation solutions developed within Horizon Europe projects and scientifically validated methodological models. I fully acknowledge the reviewer's comments regarding originality, validation, methodological transparency and the need for stronger theoretical positioning. After reflecting on the review, I have concluded that the manuscript would benefit from a fundamentally different positioning. Rather than attempting to revise it as a methodological paper, I believe it would be more appropriate to redevelop it in the future as an exploratory case study describing the stakeholder engagement practices and lessons learned from the KijaniSpace project, with the proposed framework presented as an empirical synthesis rather than as a new methodological model. I would once again like to express my sincere gratitude for the reviewer's exceptionally constructive comments. Regardless of the future of this particular manuscript, the review has provided me with valuable insights that will significantly improve both my future scientific writing and the way I position practical innovation outcomes arising from European research and innovation projects.

 Tuula

# Response

**Published:** 2026-07-22

**Authors:** Tuula-Maija Löytty

We thank Reviewer 1 for the detailed assessment and for recognising the relevance of the topic and the practical value of the KijaniSpace case. The comments identified important weaknesses in the original manuscript, particularly its claims of methodological novelty and validation. We have addressed these concerns through a substantial reframing and restructuring of the article.

**1. Originality of the framework** **Comment:** The proposed framework closely resembles existing participation frameworks, particularly the IAP2 Spectrum, and its originality and need were insufficiently demonstrated.

**Response:** We agree that the original manuscript overstated the novelty of the framework. The revised article no longer presents it as a new or validated stakeholder engagement methodology. It is now described as a retrospective analytical framework that organises documented KijaniSpace activities using concepts derived from IAP2, Agricultural Innovation Systems, living lab research and multi-actor innovation literature. The revised Chapter 4 explicitly identifies IAP2 as an important conceptual starting point and explains that the categories Inform, Consult, Involve and Collaborate were adapted for retrospective classification. It also explains why Empower was not included: the documentation did not demonstrate transfer of final decision-making authority to external stakeholders. The contribution is therefore no longer claimed to be the invention of new participation stages. It is the retrospective organisation of heterogeneous activities implemented across a Horizon Europe multi-actor project into a coherent analytical structure.

**2. Digital Agriculture and Climate-Smart Agriculture** **Comment:** Digital Agriculture and Climate-Smart Agriculture were used ambiguously or interchangeably, and the role of Space–IoT technologies was unclear.

**Response:** We have added separate definitions in the Background section. Digital Agriculture is defined as the use of technologies such as Earth Observation, IoT, sensors, artificial intelligence and digital advisory services to support agricultural decision-making and resource management. Climate-Smart Agriculture is described as an approach seeking to improve productivity, strengthen climate resilience and, where appropriate, reduce environmental impacts. The revised manuscript explains that Space–IoT technologies are enabling technologies within KijaniSpace: they provide data and digital services that may support climate-smart agricultural and aquaculture decision-making. The concepts are therefore related but not treated as synonymous.

**3. Limited empirical validation** **Comment:** Project activities and examples do not constitute validation of the framework, and no comparison with alternative approaches or outcome indicators was provided.

**Response:** We agree. The revised article no longer claims to validate the framework or demonstrate its effectiveness. It is explicitly presented as a retrospective descriptive case study and an interpretive classification exercise. The abstract, methods, results, discussion and conclusions now state that the study does not establish causal relationships between engagement activities and trust, ownership, adoption, institutional embedding, exploitation readiness or long-term sustainability. These outcomes were not measured and are identified as subjects for future longitudinal and participant-based research.

**4. Reproducibility and methodological transparency** **Comment:** More information was needed on sampling, participants, workshops, data collection and analytical procedures. **Response:** The Materials and methods section has been substantially expanded. It now identifies:

- the retrospective descriptive case-study design;
- documented stakeholder engagement activities as the unit of analysis;
- the documentary corpus, including the detailed stakeholder mapping dataset, aggregated summaries, D2.1, D2.2 and D6.2;
- the types of information extracted into the coding table: activity, primary objective, interaction format, intended stakeholder role, location and documentary source;
- the criteria used to classify activities;
- the use of dominant purpose when an activity displayed characteristics of more than one category.

The Results also provide participant or reach information where it is documented and distinguish website reach, registrations, interview participants and mapped stakeholders rather than treating them as comparable measures of engagement quality. Because identifiable stakeholder data contain names, affiliations and contact information, they cannot be publicly released. The Data availability statement now explains that aggregated distributions and public project evidence are available, while access to non-public materials is subject to data-protection requirements, consent and consortium approval.

**5. Clarity, precision and contextualisation** **Comment:** Several statements were overly broad, insufficiently sourced or unclear about their geographical and institutional context. **Response:** The manuscript has been revised throughout to specify the Horizon Europe, AU–EU and Lake Victoria Basin contexts. Chapter 3 now identifies the participating countries, project objectives, regional context, KijaniBox concept and planned stakeholder activities, supported by project, FAO, IPCC and Lake Victoria Basin sources. Generic statements have been removed, qualified or supported with references. The revised paper also avoids implying that findings from one project automatically apply to all digital agriculture or multi-actor projects.

**6. Trust, ownership, adaptive governance and sustainability** **Comment:** These concepts were not operationally defined or measured, but the manuscript presented them as outcomes. **Response:** We agree. Claims that KijaniSpace “strengthened,” “generated” or “demonstrated” trust, ownership, exploitation readiness or sustainability have been removed. The revised manuscript states that the documentation records communication, consultation, co-design and collaboration-oriented activities, but does not establish their longer-term relational or institutional effects. Where these concepts remain in the Discussion, they are presented as possible implications from the literature or as future research questions, not as empirical findings.

**7. Figure 1** **Comment:** Figure 1 was too simplistic for a methodological contribution. **Response:** Following the reframing of the article, Figure 1 is no longer presented as a validated methodological or causal model. It now serves as a visual summary of the four analytical categories and their possible relationships. The caption and surrounding text explicitly state that the arrows do not represent mandatory progression and that the horizontal dimension is conceptual rather than measured.

**8. Comparison with existing frameworks** **Comment:** A comparative table with existing stakeholder engagement frameworks would clarify the contribution.

**Response:** We have strengthened the direct comparison with IAP2 in the text, including the origin of the four categories, their analytical adaptation and the exclusion of Empower. Because the revised paper no longer claims superiority or replacement of existing frameworks, we did not add a comparative performance table. Instead, the manuscript positions the framework as an application-oriented synthesis for interpreting KijaniSpace documentation.

**9. Indicators of stakeholder progression** **Comment:** The manuscript should specify indicators used to assess progression.

**Response:** The revised manuscript does not claim to assess individual stakeholder progression. It explicitly states that the documentation did not track participant trajectories sufficiently to demonstrate movement between categories. The term progression refers only to conceptually different opportunities for interaction and contribution. Communication reach, interview participation, workshop registrations and collaboration-oriented activities are reported as descriptive evidence, not as a formal progression scale. Developing and validating participant-level indicators is proposed as future research.

**10. Self-assessment bias** **Comment:** The limitations should address bias arising from project self-assessment.

**Response:** The limitations now acknowledge that the analysis is based on documents generated through project planning, implementation and reporting rather than independent research data. The paper recognises the possible influence of selective documentation, researcher involvement and the absence of independently collected stakeholder evaluations. These limitations are also reflected in the cautious interpretation of the findings.
